# Genome-wide association study identifies 143 loci associated with 25 hydroxyvitamin D concentration

**DOI:** 10.1038/s41467-020-15421-7

**Published:** 2020-04-02

**Authors:** Joana A. Revez, Tian Lin, Zhen Qiao, Angli Xue, Yan Holtz, Zhihong Zhu, Jian Zeng, Huanwei Wang, Julia Sidorenko, Kathryn E. Kemper, Anna A. E. Vinkhuyzen, Julanne Frater, Darryl Eyles, Thomas H. J. Burne, Brittany Mitchell, Nicholas G. Martin, Gu Zhu, Peter M. Visscher, Jian Yang, Naomi R. Wray, John J. McGrath

**Affiliations:** 1 Institute for Molecular Bioscience, The University of Queensland, Brisbane, QLD Australia; 2 Queensland Brain Institute, The University of Queensland, Brisbane, QLD Australia; 3 Queensland Centre for Mental Health Research, The Park Centre for Mental Health, Wacol, QLD Australia; 4 QIMR Berghofer Medical Research Institute, Brisbane, QLD Australia; 5 School of Biomedical Sciences, Faculty of Health, and Institute of Health and Biomedical Innovation, Queensland University of Technology, Brisbane, QLD Australia; 6 Institute for Advanced Research, Wenzhou Medical University, Wenzhou, Zhejiang 325027 China; 7 National Centre for Register-based Research, Aarhus University, Aarhus, Denmark

**Keywords:** Genome-wide association studies, Calcium and vitamin D, Psychiatric disorders, Risk factors

## Abstract

Vitamin D deficiency is a candidate risk factor for a range of adverse health outcomes. In a genome-wide association study of 25 hydroxyvitamin D (25OHD) concentration in 417,580 Europeans we identify 143 independent loci in 112 1-Mb regions, providing insights into the physiology of vitamin D and implicating genes involved in lipid and lipoprotein metabolism, dermal tissue properties, and the sulphonation and glucuronidation of 25OHD. Mendelian randomization models find no robust evidence that 25OHD concentration has causal effects on candidate phenotypes (e.g. BMI, psychiatric disorders), but many phenotypes have (direct or indirect) causal effects on 25OHD concentration, clarifying the epidemiological relationship between 25OHD status and the health outcomes examined in this study.

## Abstract (web summary)

Vitamin D is a precursor of the steroid hormone 1,25-dihydroxyvitamin D3, and its deficiency is associated with many adverse health outcomes. Here, Revez et al. perform a genome-wide association study for circulating 25-hydroxyvitamin D in 417,580 individuals and test for potential causal relationships with other traits using Mendelian randomization.

## Introduction

In recent decades, there has been considerable interest in the links between vitamin D concentration and general health. While classically linked to bone disorders, there is growing evidence to suggest that suboptimal vitamin D status may be a risk factor for a much wider range of adverse health outcomes^1^. Vitamin D, the sunshine hormone, is the precursor of a seco-steroid transcription regulator that operates via a nuclear receptor, and like other steroid hormones, exerts transcriptional control over many regions of the genome across many different tissues. In environments with access to adequate sunshine, ultraviolet radiation on the skin converts a precursor of cholesterol to vitamin D_3._ This is then further converted to 25-hydroxyvitamin D_3_ (25OHD; used in assays of general vitamin D status), and then to the active hormone 1,25-dihydroxyvitamin D_3_ (1,25OHD) in a variety of tissues. Some foods and vitamin D supplements also contribute to vitamin D levels. Definitions of vitamin D deficiency (e.g., <25 nmol L^−1^ of 25OHD) are predominantly based on bone health^2^—according to these definitions, vitamin D deficiency is common in many countries, regardless of latitude and economic status^3^.

Environmental factors such as season of testing and latitude contribute substantially to the serum concentration of 25OHD (lower in winter/spring; lower at higher latitudes)^1^. With respect to the genetic architecture of 25OHD, twin and family studies have reported a wide range of heritability estimates (from 0%^4^ to 90%^5^). A recent multivariate twin study demonstrated that approximately half of the total additive genetic variation in 25OHD may reflect genetic variation in skin colour and sun exposure behaviour^6^. Genome-wide association studies (GWAS) have identified common single-nucleotide polymorphisms (SNPs) located in biologically plausible genes^7^. The largest GWAS to date (*N* = 79,366) reported six significant loci, which include *GC* (the vitamin D-binding protein gene), the *DHCR7/NADSYN1* region (*DHCR7* is involved in a conversion of a 25OHD precursor molecule to cholesterol) and *CYP2R1* and *CYP24A1* genes (which encode enzymes involved in 25OHD metabolism^8^). In total, common SNPs explain 7.5% (standard error (s.e.) 1.9%) of the variance of 25OHD^8^.

**Fig. 1:**
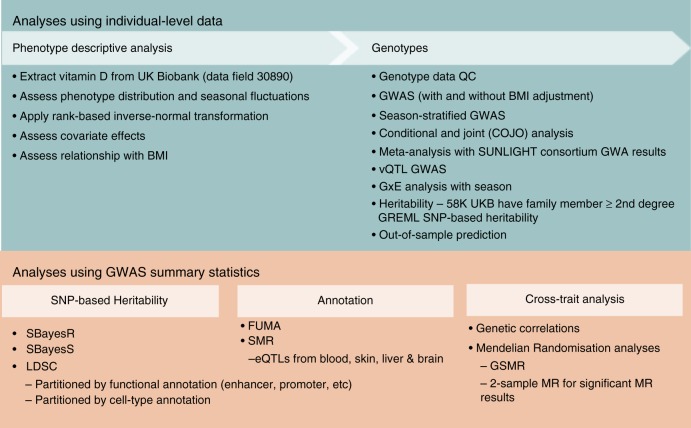
Outline of key analytic steps described in this study. BMI body mass index, eQTL expression quantitative trait locus, FUMA functional mapping and annotation^23^, GREML^69^ genomic relationship restricted maximum likelihood^68^, GSMR generalised summary-based MR, GWAS genome-wide association study, GxE genotype-by-environment interaction, LDSC linkage disequilibrium score regression, MR Mendelian Randomisation, SMR summary-based MR^26^, QC quality control, UKB UK Biobank, vQTL variance quantitative trait locus^12^.

Here, we conduct a GWAS of 25OHD based on the large UK Biobank (UKB) sample^9^ and conduct a suite of post-GWAS analyses to aid interpretation of the results (Fig. 1). We present models that explore the genetic or causal relationship between body mass index (BMI) and 25OHD (high BMI is associated with lower 25OHD concentration in observational studies^10^). Because we have an interest in the association between 25OHD and psychiatric disorders^11^, we use Mendelian randomisation methods to investigate the bidirectional association between 25OHD and psychiatric disorders, as well as with a wider range of traits and diseases. In addition, we present a GWAS to identify loci associated with variance in 25OHD (i.e., variance quantitative trait locus (vQTL) analysis) which can identify putative genotype environment interactions without prior identification of the environmental effect^12^. We identify 143 independent loci in 112 1-Mb regions associated with 25OHD concentration, and our findings implicate genes involved in lipid metabolism, dermal tissue properties and conjugation of 25OHD. We find no robust evidence that 25OHD concentration has causal effects on candidate phenotypes. However, we show that many phenotypes have (direct or indirect) causal effects on 25OHD concentration.

## Results

### 25OHD phenotype

In total, 417,580 European UKB participants had both measures of vitamin D 25OHD and genome-wide genotypes (“Methods”). The distribution of 25OHD concentration, in keeping with expectation, is right skewed (Supplementary Fig. 1a), and showed the expected seasonal fluctuation (Supplementary Fig. 1b–d), with median, mean and interquartile range of 47.9, 49.6, 33.5–63.2 nmol L^−1^ (Supplementary Table 1). Covariates of age, BMI, genotyping batch, assessment centre, month of testing, supplement intake and the first four ancestry principal components (PCs), but not sex, were all significantly associated with 25OHD (Supplementary Table 1). Month of testing accounts for 14% of the variance of 25OHD. Subsequent analyses use 25OHD after rank-based inverse-normal transformation (RINT), unless otherwise stated.

### Heritability and SNP-based heritability

**Fig. 2:**
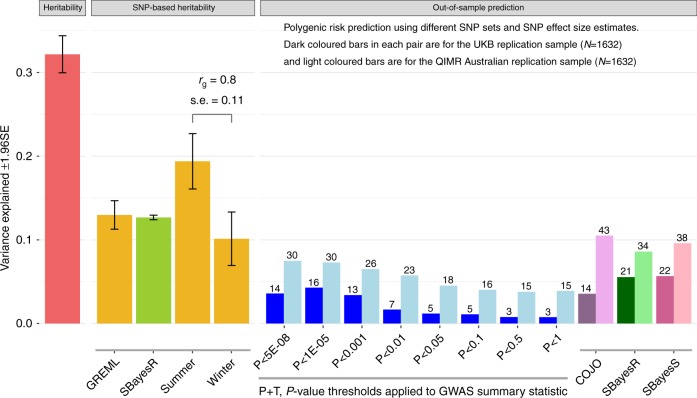
Heritability, SNP-based heritability and variance explained in out-of-sample prediction. Heritability (left panel) and SNP-based heritability (middle panel) estimates and the variance explained in out-of-sample prediction (right panel). Heritability and SNP-based heritability estimates are presented with 95% confidence interval. GCTA-GREML was used to estimate heritability from a UKB subset that included all pairs of individuals related with coefficient of relationship > 0.2 (*N* = 58,738 relatives). GCTA-GREML was used to estimate SNP-based heritabilities labelled GREML summer or winter using samples of ~50 K participants randomly drawn from the UKB. The SBayesR SNP-based heritability is estimated from the GWAS summary statistics (*N* = 417,580). In out-of-sample prediction into the QIMR and the UKB replication (UKBR) samples, polygenic risk scores (PRS) calculated by the standard *P*-value threshold method (P + T) were outperformed by using SNP effect estimates calculated from GWAS summary statistics using the SBayesR or SBayesS methods. Bars of the same colour used the same methodology (noting that SBayesR generates an estimate of SNP-based heritability as well as SNP effect sizes in prediction analysis). The numbers on top of the bars are −log10 *P*-value of the regression of 25OHD on 25OHD PRS. COJO conditional and joint, rg genetic correlation, s.e. standard error.

Our UKB sample included a set of 58,738 individuals related with coefficient of relationship (*r*) > 0.2 to at least one other person in the set (all relatives), from whom we estimate the heritability of 25OHD to be 0.32 (s.e. = 0.01) with little evidence for inflation from shared family environment (Fig. 2; Supplementary Fig. 2, Supplementary Data 1, Supplementary Note 1). The SNP-based heritability estimate ($\hat h_{{\mathrm{SNP}}}^2$), which captures the genetic contribution from common (minor allele frequency or MAF > 0.01) variants, was 0.13 (s.e. = 0.01) (see Supplementary Fig. 2, Supplementary Data 1 for a comparison of $\hat h_{{\mathrm{SNP}}}^2$ estimated from various methods). $\hat h_{{\mathrm{SNP}}}^2$ was significantly higher (*P* = 1.5 × 10^−3^; *Z*-test difference between two estimates, H0:difference = 0) when estimated only from individuals measured for 25OHD in summer months (June to October) compared with those measured in winter months (December to April) (0.19, s.e. = 0.02 vs. 0.10, s.e. = 0.02) (Fig. 2), as found for estimates of twin heritability^6^. The genetic correlation between the seasons was 0.80 (s.e. = 0.11), not significantly different from 1. The proportion of SNPs estimated to have an effect on the trait (polygenicity parameter) using the SBayesS method^13^ was 0.8% or 9000 SNPs of the ~1.1 million HapMap3 panel^14^ common SNPs (Supplementary Table 2), much lower than estimates for most complex traits^13^. The SBayesS *S* parameter, which describes the effect size–MAF relationship, was estimated as −0.78 (s.e. = 0.04; Supplementary Table 3), consistent with a model of negative selection on the genetic variants associated with 25OHD levels (the magnitude of *S* is higher than those of most complex traits studied^13^). Estimation of $\hat h_{{\mathrm{SNP}}}^2$ partitioned into ten components based on five MAF bins (each median split by linkage disequilibrium score) did not provide strong evidence for an increased role for less common variants, given the s.e. of estimates (Supplementary Fig. 3). Despite a strong phenotypic association between 25OHD and BMI of −0.76 nmol L^−1^ per BMI unit (−0.036 RINT(25OHD) standard deviation (SD) units per BMI unit, linear regression *P* < 2.2 × 10^–16^) and a phenotypic correlation of −0.17 (Supplementary Table 1), the estimates of heritability (both family and SNP-based) were hardly impacted when BMI was included as a covariate (Supplementary Fig. 2).

### Genome-wide association study (GWAS) analysis

Given the potential for collider bias from using a heritable trait as a covariate^15^, we conducted GWAS for 25OHD with and without BMI as a covariate. We also used mtCOJO^16^ to estimate the 25OHD SNP effects conditioning on those estimated for BMI from UKB data^17^, a summary-data-based conditional analysis approach that was shown in simulations to be robust to collider bias when conditioning on a correlated trait^16^. Results were comparable across the three levels of BMI adjustment (Supplementary Data 2), so we report those with no correction for BMI, using results from all three analyses when this aids interpretation of the results. While there is some debate about the clinical threshold for vitamin D deficiency (25 nmol L^−1^ or 30 nmol L^−1^ serum concentration)^1,2^, we chose the more conservative threshold of 25 nmol L^−1^. We conducted analyses bisecting 25OHD into a binary trait (less than 25 nmol L^−1^, 25 nmol L^−1^ or greater), but the results were consistent (given the expected reduced power) with our reported results treating 25OHD as a quantitative trait (Supplementary Note 2).

**Fig. 3:**
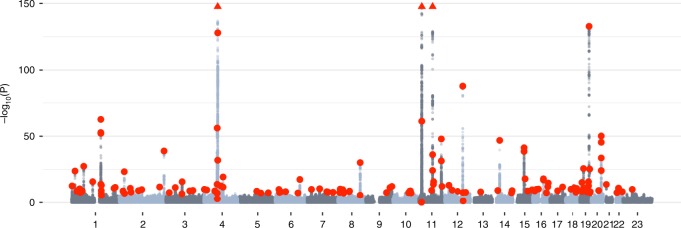
Manhattan plot of the 25OHD GWAS in the UK Biobank. Manhattan plot showing the −log10 *P*-values from the fastGWA^62^ association test of 25-hydroxyvitamin D (25OHD) with genome-wide SNPs. Red dots represent independent variants identified as genome-wide significant with conditional and joint analysis (COJO^18^) applied to the GWAS summary statistics. The horizontal axis shows each chromosome, with 23 representing the X chromosome. The vertical axis is restricted to −log10 *P*-values < 150. Five COJO SNPs were associated at *P* < 1 × 10^−^^150^ (four on chromosome 11 and one on chromosome 4; Supplementary Data 3) and have approximate locations represented by three red triangles at the top edge of the plot.

**Fig. 4:**
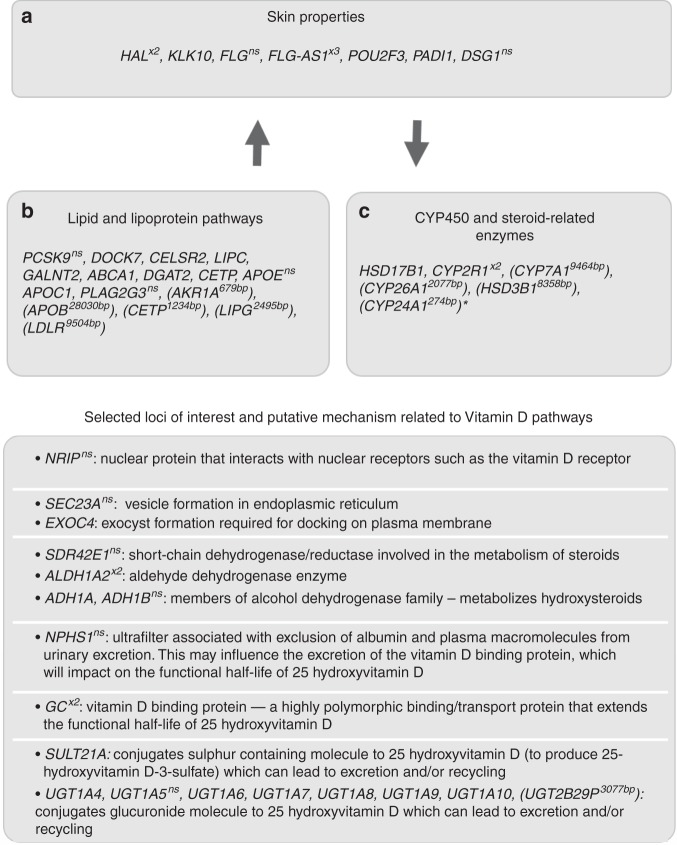
Summary of selected variants associated with 25OHD in the UK Biobank. Top panel shows loci associated with skin integrity, lipid and lipoprotein pathways and CYP450 and steroid-associated enzymes. Lower panel shows selected variants and putative mechanisms related to 25-hydroxyvitamin D (25OHD) concentration. For selected inter-genic loci, the nearest (upstream or downstream) gene is shown in brackets. The distance between the loci and the nearest gene is shown in base pairs. *CYP24A1 was also the closest gene for an additional three inter-genic loci with distances between 32,865–55,282 base pairs. ns non-synonymous variant, x2 or x3 two or three loci found within the gene. We note that nearest gene annotation should be interpreted recognising that these are not proof of a causal relationship between the associated SNP and expression of the gene (none-the-less supporting evidence for causal relationships is given by the SMR analyses for some loci).

A total of 8,806,780 SNPs with MAF > 0.01 were tested in the GWAS analysis. Of these, 18,864 were genome-wide significant (GWS; *P* < 5 × 10^−8^). To identify independently associated loci, we applied the GCTA–COJO method^18^ to the GWAS summary statistics using LD between SNPs estimated from a UKB subset (“Methods”), and identified 143 independent loci (including one on chromosome X) (Fig. 3; Supplementary Data 3) in 112 1-Mb regions. Of these, 15 loci were low-frequency variants (MAF < 0.05), and 106 regions had no previously identified associations. All six loci reported in previous vitamin D GWAS^8,19,20^ were replicated in our study. While recognising that the COJO method cannot distinguish between SNPs in perfect LD, we note that within the 143 COJO independent variants: (a) 14 were non-synonymous variants that alter protein coding (*NRIP1*, *DSG1*, *TM6SF2*, *PLA2G3*, *GCKR*, *APOE*, *PCSK9, SEC23A*, *FLG*, *NPHS1*, *SDR42E1*, *CPS1*, *ADH1B*, *UGT1A5*), and (b) 9 were annotated to include small insertion/deletions. A summary of the results is provided in Fig. 4, but are discussed later.

Summary statistics from the SUNLIGHT consortium^8^ were available for 2,579,297 SNPs, and the genetic correlation estimate with UKB results was not significantly different from 1 ($\hat r_g$ = 0.92, s.e. = 0.06). Meta-analysis with our UKB GWAS results after imputation^21^ of the SUNLIGHT summary statistics (Supplementary Methods) (6,912,294 overlapping SNPs) identified 15,154 GWS variants, 150 GCTA–COJO independent SNPs (Supplementary Methods, Supplementary Data 2). Of these, 91 were within 1-Mb regions also identified in the UKB alone as GWS. Given that the meta-analysis only increased the total number of significant loci by seven, and given our preference not to include BMI as a covariate, we continued with the UKB-only results for our downstream analyses. See Supplementary Note 3 for further details.

### Replication and out-of-sample genetic risk prediction

Of the 143 genome-wide significant COJO SNPs, 135, 135 and 108 were found in the QIMR (*N* = 1632), UKBR (*N* = 1632) and SUNLIGHT (*N* = 79,366) consortium replication samples, respectively (Supplementary Data 3; Supplementary Note 3). Of these, 92 (68%, *P* = 6.7 × 10^−6^), 97 (72%, *P* = 7.5 × 10^−8^) and 89 (82%, *P* = 5.3 × 10^−13^) had the same sign of association test statistic as in the UKB discovery sample (*P*-values from the binomial test with null hypothesis of random sign). The pairwise correlations between allele effect size estimates in the different cohorts were all highly significant, ranging from 0.44 between QIMR and UKBR and 0.91 between UKB and SUNLIGHT. Polygenic score prediction into the QIMR sample using SNP effects estimated in the UKB and the standard *P*-value thresholding method explained a maximum of 7.5% of the variance in RINT (25OHD residuals after regression on covariates) (Fig. 2; Supplementary Table 4) (linear regression *P* = 3.7 × 10^−31^, at *P*-value threshold of *P* < 5 × 10^−8^). When the polygenic scores were derived from SNP weights from COJO or SBayesR applied to the GWAS summary statistics^13,22^, the prediction variance was higher 10.5% and 9.6%, respectively (Fig. 2; Supplementary Table 4). In the UKBR sample, the *P*-value thresholding method explained a maximum of 4.3% of variance (*P* = 3.2 × 10^−17^, at *P*-value threshold of *P* < 1 × 10^−5^), while scores using the COJO SNP weights explained 3.6% of the variance (*P* = 1.6 × 10^−14^) and those using SBayesR SNP weights explain 5.5% of the variance (*P* = 5.7 × 10^−22^, Fig. 2; Supplementary Table 4).

### Functional mapping and annotation of GWAS

To annotate the 25OHD GWAS, we first used the FUMA online pipeline^23^. Gene-set analyses showed that the top four pathways were related to glucuronidation, ascorbate and aldarate metabolism and uronic acid metabolism (Supplementary Data 4, 5). Keratinisation was the top Gene Ontology (GO) biological processes identified. Based on 53 tissue types from GTEx v6^24^, the top tissues for differentially expressed genes identified in the GWAS were liver, brain and skin (sun exposed, and non-sun exposed; Supplementary Data 6). Partitioned SNP-based heritability analysis^25^ using cell-type-specific annotations identified five cell types (hepatocytes, two types of liver cells, skin cells and blood cells) at the nominal significance level of 0.05 (Supplementary Data 7), but none remained significant after correction for multiple testing (stratified LD score regression *P* (*P*_LDSC_) < 2.4 × 10^−4^). In partitioned SNP-based heritability analysis using SNP annotation to 53 functional categories^25^, 11 passed multiple testing significance threshold (*P*_LDSC_ < 9.4 × 10^−4^; Supplementary Data 8) with a mix of annotations including transcription factor binding sites and transcription start sites (notable because vitamin D operates via a nuclear receptor, which binds to vitamin D response elements), as well as a role for repressed sites, conserved regions, enhancer and coding regions and histone modification marks.

To identify 25OHD SNP associations with statistical evidence consistent with a causal/pleiotropic association via gene expression, we used summary-data-based Mendelian randomisation (SMR)^26^ using the 15,504 gene probes with significant cis-eQTLs identified from whole blood eQTLGen data^27^. After Bonferroni correction, we found 112 significantly associated gene expression probes (*P*_SMR_ < 3.2 × 10^−6^, i.e., 0.05/15,504, being the total number of probes tested in SMR analysis; Supplementary Data 9, Supplementary Fig. 4; full details of the SMR analyses can be found on https://cnsgenomics.com). These results are discussed in detail in Supplementary Note 4, and add weight to the hypothesis that the SMR-identified eQTL variants may be causally related to 25OHD concentrations.

### Putative causal relationships with other traits

First, we investigated the relationship between 25OHD and BMI. The LDSC^28^ genetic correlation estimated from 25OHD and BMI GWAS summary statistics was −0.17 (s.e. = 0.03) (Supplementary Fig. 2, Supplementary Data 10). Bidirectional Mendelian randomisation^16^ analysis provided strong support for the hypothesis that high BMI is causal for low 25OHD (*b*_BMI.25OHD_ = −0.130; s.e. = 0.005; *P*_GSMR_ = 4.7 × 10^−162^; based on 1020 BMI-associated SNP instruments), with no support for a causal effect of vitamin D on BMI (*b*_25OHD.BMI_ = 0.008; s.e. = 0.006; *P*_GSMR_ = 0.20; based on 210 vitamin D-associated SNPs) (these results were confirmed by other MR methods^29^; Supplementary Table 5). Notably, the HEIDI-outlier test in the GSMR analyses excluded 70 BMI and 67 25OHD SNP instruments, whose combination of SNP effect sizes likely reflects a pleiotropic relationship or confounding. Using the SNPs excluded by the HEIDI-outlier test, the estimates were *b*_BMI.25OHD_ = 0.17 (s.e. = 0.0182; PGSMR = 1.2 × 10^−20^) and *b*_25OHD.BMI_ = −0.15 (s.e. = 0.017, *P*_GSMR_ = 2.7 × 10^−18^). Hence, despite the clear evidence for a causal relationship between high BMI and low 25OHD, the biological relationship between these traits is more complex.

Next, we estimated genetic correlations (*r*_*g*_) between 25OHD and 746 traits with GWAS summary statistics available in LD Hub^30^, and we used LDSC to estimate *r*_*g*_ between 25OHD and 18 traits (including six psychiatric disorders) with GWAS summary statistics that are more recent than those included in LD Hub. Although many of the traits are highly correlated, we use a Bonferroni correction for 764 tests as the threshold for discussion of *r*_*g*_. The LDSC regression intercepts were close to zero, suggesting no sample overlap (Supplementary Data 10), except for LD Hub traits derived from UKB analyses, where the intercept, as expected from theory, equates to the phenotypic correlation. We found significant associations between 25OHD and a range of brain-related phenotypes (including autism spectrum disorder, intelligence^31^, major depression, bipolar disorder and schizophrenia; Supplementary Fig. 5). Notably, the most significant *r*_*g*_ were with cognitive-associated traits—for example, a negative correlation ($\hat r_g$ = −0.24, s.e. = 0.03, *P*_H0:rg=0_ = 1.6 × 10^−14^) with intelligence. There was also a significant negative *r*_*g*_ with hours spent using a computer ($\hat r_g$ = −0.22, s.e. = 0.03, *P*_H0:rg=0_ = 5.1 × 10^−15^). These findings may be mediated by an association between higher intelligence and behaviour associated with less exposure to bright sunshine (and thus, lower 25OHD). Of note, behaviours associated with outdoor activity (duration of walks, duration of vigorous activity) were positively associated with 25OHD, while phenotypes related to chronic disability were negatively associated with 25OHD.

**Fig. 5:**
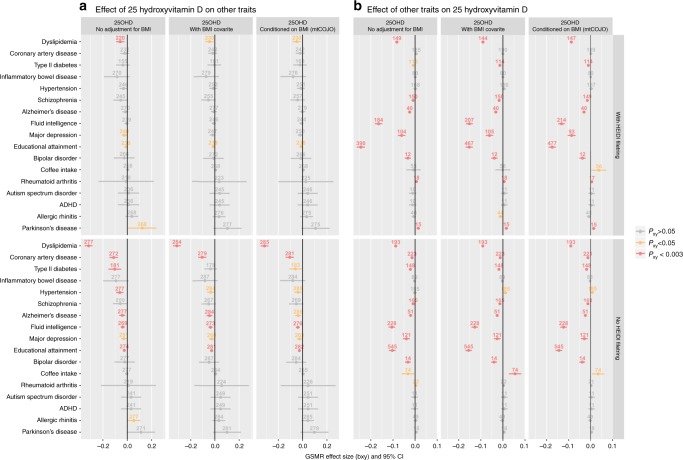
Mendelian randomisation analysis estimates from GWAS results from 25OHD and selected phenotypes. Bidirectional Generalised Summary-data level Mendelian Randomisation (GSMR) between 25-hydroxyvitamin D (25OHD) concentrations and selected phenotypes, by three types of adjustments for body mass index (BMI) and with/without HEIDI filtering of pleiotropic loci. Panel **a** shows the estimate of the causal effect estimate ($\hat b_{xy}$; dots), and 95% confidence intervals (bars), of 25OHD concentration on selected phenotypes. $\hat b_{xy}$ (and respective *P*-values) was obtained with the generalised summary-data-based Mendelian randomisation (GSMR) method^16^. Negative $\hat b_{xy}$ indicates that variants associated with increased 25OHD concentration were associated with a smaller value/reduced risk for the phenotypes of interest. Panel **b** shows the estimate of the causal effect (and 95% confidence intervals) of the same selected phenotypes on 25OHD concentration. Results are presented with (upper half) and without (lower half) filtering of pleiotropic associations with the heterogeneity in dependent instruments (HEIDI) test, respectively. The numbers above each effect size indicate the number of SNP instruments used in each analysis. For each set of analyses, we show GWAS results: (i) without adjustment for body mass index (BMI), (ii) with BMI included as a covariate and (iii) conditioned on BMI using mtCOJO^16^. The GMSR estimates and 95% confidence intervals are shown in three colours according to the *P*-value thresholds: grey (non-significant; PGSMR > 0.05), yellow (nominally significant; PGSMR < 0.05) and red (significant after Bonferonni correction for multiple testing; PGSMR < 0.003). All $\hat b_{xy}$, respective standard errors and *P*-values can be found in tabular form in Supplementary Data 11.

Next, we investigated if some of the significant genetic correlations could be explained by causal relationships using bidirectional GSMR models—here a more complex pattern of association emerged (Fig. 5; Supplementary Data 11). We found no evidence for putative causal effects between 25OHD and other traits; GSMR analyses without the HEIDI-outlier filtering step (Fig. 5a) suggest strong pleiotropy for some traits, such as dyslipidemia, coronary artery disease, intelligence and educational attainment. Finally, we examined the reciprocal relationship—if variants associated with a range of traits were directionally associated with 25OHD. Regardless of the use of HEIDI filtering, and often regardless of adjustments for BMI, we found evidence consistent with increased risk of several traits or disorders being causal (directly or indirectly) with lower 25OHD concentrations (Fig. 5b). This was the case for intelligence, dyslipidemia, major depression, bipolar disorder, type 2 diabetes and schizophrenia. The findings might suggest these traits or disorders are associated with behaviours that lead to reduced production of 25OHD (e.g., less outdoor activity and physical activities). The GSMR findings were also checked with the portfolio of MR methods implemented in the two-sample MR (2SMR) software^29^ (Supplementary Data 12).

### Gene–environment interplay

We conducted a genome-wide vQTL analysis, as implemented in OSCA^12^ to identify SNPs associated with variance in 25OHD (not RINT-transformed). Such associations can reflect genotype-by-environment interaction in the absence of measurement, or indeed knowledge, of the interacting environmental risk factor^12^. Using data from 318,851 unrelated individuals of European ancestry, we tested 6,098,063 variants with MAF > 0.05, and identified 4008 GWS vQTLs, of which 25 were independent (LD *r*^2^ < 0.01, 5-MB window), and several were in genes with previously described links to vitamin D-related pathways (e.g., *GC*, *UGT2B7*, *SEC23A*, *SULT2A1*, *KLK10, NADSYN1*). Of the 25 independent vQTLs, 23 were also QTLs (identified as genome-wide significant in the GWAS analysis) while the two non-QTL loci were still associated at *P*_GWAS_ < 10^−5^ (Supplementary Data 13). One was in the *POR* gene, which encodes a cytochrome p450 oxidoreductase that donates electrons from NADPH to cytochrome P450 enzymes (encoded by *CYP450* genes), which are involved in vitamin D metabolism^32^. Variants in *POR* have previously been associated with coffee intake^33^. The other exclusive vQTL (rs1030431) is 12,126 bp upstream from *UBXN2B*; the SNP is significantly associated with gall bladder diseases and lipid metabolism traits in the UKB^34^.

An environmental factor with known association with 25OHD is the season of testing. To investigate if the associations between the vQTLs and the phenotypic variance of 25OHD reflected gene–environment (GxE) interactions with season of blood draw, we performed a GxE analysis with season (winter vs. summer). Of 6,098,063 variants tested (MAF > 0.05), 1127 had a GWS (*P* < 5 × 10^−8^) interaction with season, and 1120 (99%) were also GWS in the vQTL analysis. From the 1127 GWS interactions, five were independent (LD *r*^2^ < 0.01, window 5 Mb) and were located in regions that have well-known vitamin D-related genes in chromosomes 11 (e.g., *CYP2R1* region) and 14 (e.g., *SEC23A*) (Supplementary Data 14). Notably, of the 20 vQTL loci without significant GxE with season, at least half showed no evidence at all for GxE with season (Supplementary Fig. 6), so these variants are candidates for GxE with other environmental factors.

## Discussion

We have identified 143 loci associated with 25OHD concentration. Recognising that only six associated loci had been reported to date, these discoveries provide important insights into previously unknown or poorly understood vitamin D-related pathways, and substantially increase our knowledge of the genetic correlates of 25OHD compared with previous studies^7^ (Fig. 4). First, the three most associated loci, all identified in previous studies^8^, are noteworthy (chr4:rs1352846, chr11:rs116970203 and chr11:rs12794714, all with association test *P* < 1.0 × 10^−400^, all with their minor allele reducing 25OHD). rs1352846 (MAF = 0.29 (G)) is in the *GC* locus^8,20^, which encodes a protein synthesised in the liver that binds to, and transports vitamin D and its metabolites. rs116970203 is a low-frequency variant (MAF = 0.03 (A)) located in intron 11 of the *PDE3B* gene. It is also a perfect proxy for rs117913124 (LD *r*^*2*^ = 1), a low-frequency synonymous coding variant in *CYP2R1*, which was previously reported to associate with 25OHD^19^. Another *CYP2R1* synonymous variant was also identified (rs12794714; MAF = 0.42 (A)). *CYP2R1* encodes a crucial hepatic enzyme involved in the hydroxylation of vitamin D to 25OHD. Given the complexity of the association pattern observed in chromosome 11, we confirmed the independence of the COJO identified variants using individual-level data (Supplementary Table 6). In line with previous findings^19^, the two-way conditional analysis showed that the effect of the low-frequency SNP (rs116970203 or rs117913124) and common SNP (rs12794714 or rs10741657) were largely independent.

Our findings provide convergent evidence that genes related to lipid- and lipoprotein-related pathways influence 25OHD concentration. In particular, we confirm a unidirectional relationship between SNP instruments that influence higher BMI and lower 25OHD concentration, but not the reciprocal relationship. This relationship exists against a background of a highly intercorrelated pattern of relationships between genes that influence both 25OHD and a wide range of lipid-related metabolic phenotypes. There were variants within genes with well-described functions related to lipid and lipoprotein-related pathways^35^ (e.g., *PCSK9, DOCK7, CELSR2*, *GALNT2*, *ABCA1, DGAT2*, *CETP, APOE, APOC1, PLA2G3*). In addition, several inter-genic loci had closest upstream or downstream genes of interest to lipid and lipoprotein pathways (*AKR1A, APOB, CETP, LIPG, LDLR*). Variants in these genes influence overall lipid concentrations, including the concentration of 7-dehydrocholesterol in the skin. We identified a locus (chr11:rs12803256) in an uncharacterised RNA gene (*FLJ42102*) 11,057 base pairs upstream from *DHCR7*. This region has been identified in previous GWAS studies, and *DHCR7* is a strong candidate gene because of its known role in the conversion of 7-dehydrocholesterol in the skin to pre-vitamin D_3._ We note that the broad region on Chr11 containing *DHCR7* and *NADSYN1* included several loci of interest according to both GCTA–COJO and SMR analyses—this complex area warrants additional research.

The GWAS uncovered a range of previously unreported findings, indicating that properties of the skin not related to pigmentation are associated with 25OHD concentration. While it is well known that individuals with darker skin tend to have lower 25OHD (related to the melanin content in the skin blocking UVB)^1^, our findings provide evidence that SNPs associated with genes that influence dermal development (e.g., *PADI*)^36^ and integrity (e.g., *FLG*; *FLG-AS1, POU2F3, KLK10, DSG1*)^37,38^ are also associated with 25OHD status. It has been suggested that variants in the *FLG* gene may have evolved in order to optimise 25OHD production at high latitude^39,40^. *HAL* (histidine ammonia-lyase) codes for an enzyme that deaminates L-histidine to trans-uronic acid. The top SNP in this region (rs10859995) is within an intron of this gene. The gene is expressed in the skin, and is upregulated during keratinocyte differentiation^41^. It has been demonstrated that trans-urocanic acid in the stratum corneum can absorb UVB^42^ and can reduce the production 25OHD^43^. The MAGMA gene-set analysis^23^ also showed that variants associated the uronic acid pathways were significantly overrepresented in our findings (Supplementary Data 5). The concentration of trans-uronic acid varies widely between individuals^43,44^, but is not related to skin colour/pigmentation^44^. It is important to note that our sample was restricted to Europeans, and analyses included 40 ancestry PCs as covariates, four of which were strongly associated with 25OHD (Supplementary Table 1). If these PCs capture variants related to skin colour within Europeans, these variants are less likely to be identified in our analyses. FUMA analyses did not identify an overrepresentation of variants known to be related to skin colour in our GWAS.

Our study expands the range of enzymes implicated in the synthesis and breakdown of vitamin D-related molecules. These include genes from the hydroxysteroid 17-beta dehydrogenase family (*HSD17B1*, *HSD3B1*), a family of short-chain dehydrogenases/reductases, which are involved in steroidogenesis and steroid metabolism. *CYP2R1* is a key regulator of 25OHD status, via hepatic conversion of vitamin D to 25OHD—two loci were found within this gene. Other members of this large family of enzymes associated with 25OHD concentrations include *CYP7A1*, *CYP26A1* and *CYP24A1*.

We identified many variants within genes related to the modification of lipophilic molecules (including seco-steroids, such as 25OHD and related species). Associated regions on chromosomes 2 and 4 include enzymes in the UDP-glucuronosyltransferase family, which are critical in the glucuronidation pathways. The involvement of these genes in the degradation and potential conjugate recycling of 25OHD has recently been described^45,46^. We identified variants in the *SULT21A* gene, which encodes the enzyme responsible for the sulphonation of 25OHD^46,47^. Our findings provide support for the hypothesis that these mechanisms influence 25OHD concentration. We identified variants in the *SLCO1B1* gene, which encodes a transmembrane receptor that mediates the sodium-independent uptake of numerous endogenous compounds, including sulfated steroid molecules^48^. It is not known if this mechanism is involved in the uptake of the sulphated 25OHD. It has been proposed that vitamin D may undergo conjugate cycling (e.g., bidirectional conversion between 25OHD and 25OHD-sulphate)^49^. A proportion of the total 25OHD may exist in the sulphated form, which could act as circulating reservoir for later de-sulfation in peripheral tissues. In addition, conjugated versions of 25OHD with glucuronide^45^ and sulfate^46^ have both been detected in bile, which suggests enterohepatic mechanisms may provide another reservoir that buffers total 25OHD reserves. The findings also have implications for how to assay total 25OHD reserves. Current extraction and assay techniques used to quantitate 25OHD are not optimised for sulphonated or glucuronidated species of 25OHD, thus total 25OHD status may not accurately reflect the contribution of these conjugated species. In addition, these mechanisms would contribute to the functional half-life of 25OHD, and thus influence vitamin D status during periods of reduced exposure to bright sunshine (e.g., during winter). Finally, variants in a range of previously unreported enzymatic pathways were also associated with 25OHD concentration (e.g., short-chain dehydrogenase/reductase, aldehyde dehydrogenase, alcohol dehydrogenase).

The large sample size afforded by the UKB sample provides a thorough description of the genetic architecture of 25OHD. The SNP-based heritability (which captures the contribution to variation between people associated with common DNA variants) estimated in the UKB was 0.13 (95% CI: 0.12–0.14), which means that all genotyped/imputed variants with MAF > 0.01 explain about 41% of the heritability estimated from close relatives (i.e., 0.13/0.32, SNP-based heritability/heritability, Fig. 2). The 143 loci represent only 112 1-Mb regions, with six of the 1-Mb regions harbouring four loci each. The final set of 143 loci was achieved by applying the COJO (conditional and joint) algorithm onto the GWAS summary statistics using the linkage disequilibrium structure to account for the correlation structure between SNPs. Two regions on chromosome 11 are particularly complex, Supplementary Data 3). Polygenic score predictor using SNP effect weights estimated in the UKB explained up to 10.5% and 5.7% of variance (after accounting for covariates) in independent samples QIMR and UKB. Aside from sampling differences, the higher variance explained in the Australian QIMR sample is in line with the higher heritability estimated from family data (QIMR 0.50 (95% CI: 0.38–0.64) vs 0.32 (95% CI: 0.30–0.34) estimated in the UKB. One explanation for the difference is that the QIMR samples were predominantly recruited at latitude 27° S; at this latitude, there is sufficient UVR to allow for vitamin D synthesis throughout the year.

We also identified 25 independent SNPs associated with variance in 25OHD—these are putative GxE loci. While five of these have strong evidence of interacting with season of measurement, at least ten are GxE candidates with yet-to-be-identified environmental risk factors, and search of published GWAS results for association with these SNPs (i.e., PheWAS^34^) may help with this prioritisation (Supplementary Data 13). In summer months, the mean 25OHD concentrations are higher, and a larger proportion of the variance could be attributed to genetic factors in summer compared with winter (SNP-based heritability of 0.19, s.e. = 0.02, vs 0.10, s.e. = 0.02, *P*_different_ = 1.5 × 10^−3^), and this reflected an increase in genetic variance rather than a decrease in residual variance (Supplementary Data 1). However, the genetic correlation from summer and winter SNP effect sizes was not significantly different from 1. Five loci were identified as significant in GxE analysis with season, and for two the direction of effect was reversed (Supplementary Data 14). The vitamin D phenotype is an interesting one to explore from the perspective of GxE as seasonal fluctuations provide a natural experiment to dissect components of the genetic architecture that influence synthesis (i.e., inflow) and excretion (i.e., outflow) of 25OHD-related pathways.

In the UKB participants, high BMI is associated with reduced 25OHD concentration, in keeping with a large body of observational epidemiology^50^. However, we did not find statistical evidence in support of a causal role for the 25OHD level on BMI. In contrast, there was evidence for pleiotropic effects of SNPs on the two traits as well as for high BMI being causal (directly or indirectly) for low 25OHD. Genetic correlations were significant between 25OHD concentration and a range of phenotypes (Fig. 5). However, in robust directional models, we found no evidence in support of a causal role for 25OHD concentration on these traits. Of interest, we found evidence that higher intelligence and an increased risk of several psychiatric disorders may cause reduced 25OHD concentrations. With respect to intelligence, this would be consistent with previous links between intelligence and years of education leading to working indoors, and subsequent lower concentrations of 25OHD^51^. One of our motivations for undertaking this study was to investigate the hypothesis of a causality relationship between 25OHD and psychiatric disorders^52^. The Mendelian randomisation analyses conducted here do not support a causal role for 25OHD levels and these disorders, and hence the reported epidemiological associations could reflect confounding and/or reverse causation. Vitamin D deficiency is common in those with established psychiatric disorders, as a consequence of reduced outdoor behaviour^53^. It is feasible that the observed association between 25OHD concentration in blood spot samples taken at birth with later-life increased risk of schizophrenia^11,54^ could be confounded by outdoor behaviour of mothers, which may be correlated with the mother’s genetic liability to schizophrenia. While we find no evidence to support the hypotheses that variants associated with low 25OHD concentrations were associated with any of the selected phenotypes, we note that there is a linearity assumption in our Mendelian randomisation analyses. In other words, if only very low concentrations of 25OHD are associated with adverse outcomes, then this non-linear exposure-risk association may not be confidently detected (see Supplementary Note 2 for further discussion). As the genetic architecture of the broad range of vitamin D-related phenotypes becomes better understood, issues related to potential threshold effects (e.g., disease-specific thresholds for clinical deficiency) should be re-examined.

We have identified 143 loci associated with 25OHD concentration, and have provided new directions for vitamin D research. In particular, our findings suggest that pathways related to sulphonation and glucuronidation warrant closer scrutiny—for example, there may be a case to measure these modified species of 25OHD and related molecules in order to better understand vitamin D status. Our studies based on Mendelian randomisation do not support hypotheses that vitamin D concentration is associated with a broad range of candidate phenotypes, in particular, psychiatric disorders. The findings provide insights into the physiology of vitamin D and the relationship between 25OHD status and health.

## Methods

### The UK Biobank sample

The UK Biobank (UKB) is a large population cohort with phenotype, genotype and clinical information on more than 502,000 individuals (age range from 40 to 69 years old). Participants were registered with the National Health Service, and lived ~25 miles from one of the 22 recruitment centres across the UK^9^. Participants were recruited between 2006 and 2010. Informed consent was obtained by UK Biobank from all participants, and the study was approved by the North West Multicentre Research Ethnics Service Committee. The participants of the study were not representative of the original sampling frame, with evidence of a healthy volunteer bias^55^.

Genotype data were quality-controlled and imputed to the Haplotype Reference Consortium (HRC)^56^ and UK10K^57^ reference panels by the UKB group^58^. We extracted variants with minor allele count (MAC) > 5 and imputation score >0.3 for all individuals, and converted genotype probabilities to hard-call genotypes using PLINK2 (--hard-call 0.1)^59^. Then, we excluded variants with genotype missingness >0.05, Hardy–Weinberg equilibrium *test P* > 1 × 10^−5^, and minor allele frequency (MAF) < 0.01. In total, 8,806,780 variants (hereafter SNPs, but could include small insertion/deletions (INDELS)), including 260,713 SNPs in the X chromosome, were available for analysis.

Individuals of European ancestry were identified by projecting the UKB sample to the first two principal components (PCs) of the 1000 Genome Project (1KGP^60^), using Hap Map 3 (HM3) SNPs with MAF > 0.01 in both data sets. European ancestry was assigned based on >0.9 posterior probability of belonging to the 1KGP European reference cluster.

### Assessment of 25-hydroxyvitamin D concentration

Vitamin D 25OHD levels were measured in blood samples collected at two instances: the initial assessment visit, conducted between 2006 and 2010, and a repeat assessment visit, conducted between 2012 and 2013. The Diasorin Liason®, a chemiluminescent immunoassay (CLIA) was used for the quantitative determination of 25OHD. The assay measures total 25OHD concentration (i.e., 25OHD_3_ and 25OHD_2_). Participants with 25OHD concentrations below or above the validated range for the assay (10–375 nmol L^−1^) were excluded. The average within-laboratory coefficient of variation (CV) (and standard deviation) ranged from 5.04 (4.73) to 6.14 (2.21)^61^.

Of 502,536 UKB participants, 449,978 (90%) had vitamin D 25OHD levels (data field 30890) measured, mostly from the initial assessment visit (448,376, 99.6%). Our analyses were limited to the 417,580 individuals of European ancestry with 25OHD concentrations available, of whom 318,851 are unrelated (gcta --rel-cut-off 0.05).

### Genome-wide association study (GWAS) analysis

Figure 1 provides a graphical summary of the GWAS and post-GWAS analyses detailed below. To identify genetic variants associated with 25OHD levels, we performed a linear mixed model GWAS implemented in fastGWA^62^. fastGWA is a tool implemented in GCTA^63^ for mixed linear model (MLM)-based GWAS. It uses a sparse genomic relationship matrix (GRM) to account for genetic structure within the cohort, making it a resource-efficient method for the analysis of large data sets like the UK Biobank^62^. The sparse GRM was generated for UKB individuals of European ancestry using HapMap3 SNPs.

We applied a rank-based inverse-normal transformation (RINT) to the phenotype (vitamin D 25OHD levels) and fit age at time of assessment, sex, assessment month, assessment centre, supplement-intake information, genotyping batch and the first 40 ancestry PCs as covariates in the model (see Supplementary Methods for more details).

To identify independent associations, we a conducted a conditional and joint (COJO; gcta --cojo-slct) analysis^18^ of the GWAS results, accounting for the correlation structure between SNPs within a 10-Mb window (COJO default parameter) and using a random subset of 20,000 unrelated Europeans from the UKB as linkage disequilibrium (LD) reference. For comparison, we used PLINK1.9 (--clump)^64^ to identify regional lead SNPs for genome-wide significant index variants (--clump-p1 5e-8) and variants were clumped with this lead SNP if they were located less than 10 Mb (--clump-kb 10000) away from, and with *r*^2^ > 0.01 (--clump-r2 0.01) with the index variant. To identify previously unreported associations, we a conducted a COJO analysis that conditioned (gcta --cojo-cond) on the six loci previously reported as genome-wide significant (rs2282679, rs10741657, rs12785878, rs10745742, rs8018720, rs6013897)^8,20,65^.

### Meta-analysis

The largest GWAS for 25OHD to date, from the SUNLIGHT consortium^8^, used BMI as a covariate, hence we also generated UKB results including BMI in the model and used those for meta-analysis. In addition, the UKB GWAS results used for meta-analysis differed from the reported GWAS results in that 25OHD levels were natural-log transformed, and supplement intake was not included as a covariate. Before meta-analysis, we imputed the SUNLIGHT summary statistics (2,579,297 SNPs) with ImpG^66^. After data management, we used a sample size-based approach^67^ to perform the meta-analysis (Supplementary Methods) on 6,912,294 SNPs that were shared between the data sets.

### Relationship between vitamin D and body mass index traits

High BMI is associated with lower concentrations of 25OHD^10^. For this reason, previous GWAS of 25OHD have included BMI as covariate in their analyses^8^. However, given that BMI is a highly heritable trait, covariate adjustment can induce collider bias^15^ and affect downstream analyses. To better understand the relationship between 25OHD and BMI, we estimated the phenotypic and genetic correlation between them and used generalised summary-data-based Mendelian randomisation (GSMR)^16^ to test for statistical evidence for putative causal effects between the two traits. We confirmed through simulation that the MR regression statistic is not biased by sample overlap (Supplementary Note 5). SNP instruments were selected with the default settings of the built-in GSMR clumping step (which is less stringent than used in our clumping protocol because GSMR accounts for residual correlation between SNP instruments). In addition, we conducted a multi-trait conditional and joint (mtCOJO) analysis^16^ to condition the 25OHD GWAS results on BMI GWAS summary statistics generated with the UKB^17^, an approach that was shown in simulations to be robust to induced collider bias when conditioning on a correlated trait^16^. A random subset of 20,000 unrelated individuals of European ancestry from the UKB was used as LD reference in the mtCOJO analysis.

### Heritability and SNP-based heritability

Our UKB sample included a set of 58,738 individuals who were related with coefficient of relationship (*r*) > 0.2 to at least one other person in the set (all relatives). Among these, there was a set including all pairs with 0.4 < *r* < 0.6 (1st degree), and a set including all pairs with 0.2 < *r* < 0.3 (2nd degree). We used these sets to estimate heritability of RINT(25OHD) levels (gcta --reml). To estimate SNP-based heritability, we drew a random subset (*N* ~ 50,000), selected so that no pair of individuals had *r* > 0.05. We used a model that fits a single random genetic effect with a single genomic relationship matrix (GRM) constructed from all SNPs^68^, and also a GREML-LDMS model^69^ that fits ten random genetic effects and hence ten GRM (gcta --reml --mgrm). The ten GRM were constructed from SNPs annotated to five MAF (0.01–0.1, 0.1–0.2, 0.2–0.3, 0.3–0.4 and 0.4–0.5) bins each divided into two by median LD score of the SNPs within the bin. The LD score of a SNP is a measure of the common genetic variation tagged by a SNP. The sum of the estimates for each MAF-LD bin is an estimate of the total SNP-based heritability. Under a neutral model, each of the five MAF bins is expected to explain 20% of the variance. Analyses were conducted with and without BMI as a covariate, and genetic correlation between 25OHD and BMI was estimated in a bivariate GREML analysis (gcta --reml-bivar). In addition, we estimated the genetic correlation and the genetic variance explained by 25OHD levels assessed in summer and winter (see definitions in vQTL and seasonal analysis section below), using bivariate GREML. Heritability and SNP-based heritability estimated as part of the GWAS analysis using fastGWA are also reported. Finally, we estimated SNP-based heritability by LD score regression^28^ (software default settings for European ancestry samples), SBayesR^13^ and SBayesS^13^ from GWAS summary statistics. From SBayesS, we also estimate the polygenicity (π) and selection (S) parameters.

### Replication and out-of-sample genetic risk prediction

We used the QIMR Brisbane-based twin and family sample (*N* = 1632 unrelated)^6^ for replication analyses. Samples were collected between May 1992 and January 2014, mostly from South–East Queensland (latitude 27° S). At this latitude, there is sufficient UVR to allow for vitamin D synthesis throughout the year^70^. Legal guardians gave written, informed consent prior to inclusion and testing. Studies were approved by the Human Research Ethics Committee of the QIMR Berghofer Medical Research Institute. Additional details of this study are provided elsewhere (25OHD assay methods^6^ and genotyping on HumanCoreExome-12v1-0_C or IlluminaHuman610W-Quad bead chip and quality control^71^). Genotypes were imputed to phase 3 version 5 of the 1000 Genomes Build37 (hg19)^21^. The phenotype analysed was RINT(25OHD) pre-regressed on sex, age, month of collection, ten ancestry PCs and imputation batch. Association analysis was conducted in PLINK for the genome-wide significant COJO SNPs from the UKB analysis. We tested for the same sign of effect size between the UKB and the QIMR results. Next, using the sample, we conducted polygenic score analyses. Using only SNPs present in the Brisbane cohort, we selected independently associated SNPs from the UKB cohort in order to conduct standard *P*-value thresholding PRS analysis, choosing a range of *P*-value thresholds (*P* < 5 × × 10^−8^, *P* < 1 × 10^−5^, *P* < 0.001, *P* < 0.01, *P* < 0.05, *P* < 0.1, *P* < 0.5, *P* < 1) and calculating polygenic scores for each individual in the QIMR cohort. We also calculated polygenic scores from SNP weights estimated by COJO^72^, SBayesR^22^ and SBayesS^13^. The Bayesian methods better account for the complex relationship between strength of association of, and correlation between, SNP effect sizes. For each set of polygenic scores, we estimated the proportion of variance explained by the scores in linear regression. We repeated these analyses in a sample of 1632 individuals selected from the UKB, whose PC1 value was just outside our cut-off for white European (referred to as the UKBR sample; Supplementary Methods).

### Functional mapping and annotation of GWAS

We conducted a number of analyses to annotate the 25OHD GWAS results. First, we used the FUMA online pipeline^23^ to obtain gene-based, gene-set and tissue-specific annotations. Second, we used functional annotations provided in the LDSC software to partition SNP-based heritability into 53 functional categories^25^. Annotations included elements, such as UCSC, UTRs, promoter and intronic regions, conserved regions and functional genomic annotations constructed using ENCODE^73^ and Roadmap Epigenomics Consortium data^74^. Third, we assessed the SNP-based heritability enrichment associated with different cell types. Specifically, we applied LDSC analysis to the GWAS summary statistics using scores associated with cell-type-specific expression (as provided in the LDSC software)^75^.

To help prioritise putative causal genes with expression underlying 25OHD levels, we used the summary-data-based Mendelian randomisation method (SMR)^26^. SMR integrates GWAS and eQTL (expression quantitative trait loci, SNPs associated with gene expression) results with the aim of identifying pleiotropic or causal associations between a trait of interest and gene expression. We used eQTLs derived by the eQTLGen consortium from gene expression in whole blood^27^, using the a large sample for blood eQTLs (*N* = 31,684), identifying 15,504 genome-wide significant eQTLs. In general, SNPs controlling variation in one tissue are found to control variation in other tissues^76^, hence using a large eQTL dataset is the most powerful approach. Moreover, blood is a relevant tissue for vitamin D-related gene transcription^77^. Other relevant tissues are the liver, skin and, given our hypotheses about the relationship between 25OHD and psychiatric disorders, the brain. To capture tissue-specific eQTLs in these tissues, we used GTEX eQTL data sets, despite the fact that these data sets are much smaller than the eQTLGen sample (sun-exposed skin, *N* = 369; non-sun-exposed skin, *N* = 335; liver, *N* = 153; 16 brain regions, *N* between 80 and 154). In addition, we used eQTLs identified in pre-frontal cortex (*N* = 1866) from the PsychENCODE project^78^, and foetal brain samples (*N* = 120) from O’Brien et al.^79^. SMR significant results were declared at *P*_SMR_ < 0.05/M per tissue, where M is the number SMR tests performed (i.e., the number of gene probes tested for the tissue, Supplementary Data 9). While significant SMR test results implicate a role for the eQTL gene, SNPs passing the conservative SMR heterogeneity in dependent instruments (HEIDI) test (*P*_HEIDI_ > 0.05) have robust support for the direct causal or pleiotropic relationships of the trait-associated SNPs influencing gene expression.

### Genetic correlations and putative causal relationships with other traits

Published epidemiological studies have provided an extensive set of hypotheses about causal relationships between vitamin D and a range of phenotypes^80^, including psychiatric and brain-related disorders^81^ including intelligence^31^. To characterise the relationship between vitamin D and psychiatric traits, we conducted two sets of analyses. First, we used bivariate LD score regression^28^ to estimate the genetic correlation between vitamin D and psychiatric traits using the GWAS summary statistics generated with the UKB dataset and GWAS summary statistics that are available for attention deficit/hyperactivity disorder (ADHD)^82^, Alzheimer’s disease (AD)^83^, major depression (MD)^84^, schizophrenia (SCZ)^85^, bipolar disorder (BIP)^86^ and autism spectrum disorder (ASD)^87^. In addition, we obtained genetic correlation estimates between vitamin D and 746 traits available through the LD Hub database^30^. Second, we conducted generalised summary Mendelian randomisation (GSMR) analyses^16^ to assess if there was any statistical evidence that observed correlations could be explained by a causal relationship for seventeen traits (Fig. 5). GSMR analyses were conducted as described above (see “Relationship between vitamin D and body mass index traits” section), with significance declared at 0.05/18 = 0.003. For any significant associations observed with GSMR, we confirmed our conclusions using the two-sample MR (2SMR) method^29^, which implements a range of MR models that can adjust for the potential influence of pleiotropy (MR Egger, weighted mean, inverse variance weighted, simple mode and weighted mode).

### Proxy-environment vQTL and seasonal analysis

25OHD concentration is known to be affected by season of measurement, but other environmental factors may also impact 25OHD measures. We conducted a genome-wide vQTL analysis^12^, an approach to detect presence of genotype-by-environment interaction in the absence of measurement or knowledge of the interacting environmental risk factor, to identify SNPs associated with variance in 25OHD. Specifically, we used the Levene’s median test implemented in OSCA^88^. Following the guidelines of Wang et al.^12^, we (1) adjusted 25OHD levels for selected covariates (see below), (2) removed outliers more than 5 SD from the mean and (3) standardised the residuals to have mean 0 and variance 1. Each step was performed within one of eight groups defined based on sex (male vs. female) and supplement intake (none, other, vitamin D or missing). This approach removed both the mean effect of covariates and the mean and variance differences between gender and supplement- intake groups, while retaining other distributional properties of the measure. Covariates included in the phenotype pre-regression were age at assessment, assessment month, assessment centre, genotyping batch and the first 40 PCs. To avoid spurious associations due to coincidence of low-frequency variants with phenotype outliers^12^, this analysis was restricted to SNPs with MAF > 0.05. To identify near-independent vQTLs, we clumped the vQTL GWAS results with PLINK1.9 (--clump) as above, using a 5 Mb window as recommended^12^.

To assess if significant vQTL associations reflected a GxE with season of testing, we conducted season-stratified GWAS and compared the results with the vQTL GWAS results. Specifically, we stratified the UKB cohort into two groups after visual inspection of the mean 25OHD concentrations per month (Supplementary Fig. 1b). We defined two discrete time periods in order to retain the maximum sample size, but optimise comparisons between months with higher and lower mean 25OHD concentrations: (a) Winter—individuals assessed Dec-Apr (N = 162,591), and (b) Summer—individuals assessed Jun-Oct (N = 177,082). Individuals with vitamin D levels assessed in the months of May and November were not included in these analyses. The two season-stratified GWAS (winter and summer) were conducted as the main GWAS (i.e., linear mixed model implemented in fastGWA, with the same covariates included in the model). In addition, we conducted a GxE analysis using PLINK1.9^59^ (--gxe) to include an interaction term with season of blood draw. For this analysis, the phenotype (25OHD levels) was processed as described for the vQTL analysis.

### Reporting summary

Further information on research design is available in the Nature Research Reporting Summary linked to this article.

## Supplementary information

Supplementary Information

Description of Additional Supplementary Files

Supplementary Dataset 1

Supplementary Dataset 2

Supplementary Dataset 3

Supplementary Dataset 4

Supplementary Dataset 5

Supplementary Dataset 6

Supplementary Dataset 7

Supplementary Dataset 8

Supplementary Dataset 9

Supplementary Dataset 10

Supplementary Dataset 11

Supplementary Dataset 12

Supplementary Dataset 13

Supplementary Dataset 14

Reporting Summary

## Data Availability

Genome-wide association summary statistics generated with the three levels of BMI correction (i.e., with and without BMI as covariate, and conditioned on BMI) are available for download from https://cnsgenomics.com/content/data. Results for the UKB GWAS of BMI used for conditional analysis are also available from the same website. UK Biobank data were obtained through direct application to the UK Biobank. The SUNLIGHT data were downloaded from https://drive.google.com/drive/folders/0BzYDtCo_doHJRFRKR0ltZHZWZjQ. Functional annotations to partition SNP-based heritability with LDSC were downloaded from https://data.broadinstitute.org/alkesgroup/LDSCORE/. eQTL data were downloaded from http://www.eqtlgen.org/cis-eqtls.html and https://cnsgenomics.com/software/smr/#DataResource. GWAS summary statistics used for bidirectional GSMR were downloaded from https://walters.psycm.cf.ac.uk (schizophrenia), https://cnsgenomics.com/content/data (type II diabetes), https://ctg.cncr.nl/software/summary_statistics (Alzheimer’s disease; fluid intelligence; ADHD), https://www.thessgac.org/data (educational attainment), https://www.med.unc.edu/pgc/download-results/ (bipolar disorder; autism spectrum disorder), http://plaza.umin.ac.jp/~yokada/datasource/software.htm (rheumatoid arthritis), ftp://ftp.ebi.ac.uk/pub/databases/gwas/summary_statistics/vanderHarstP_29212778_GCST005194 (coronary artery disease), https://www.ibdgenetics.org/downloads.html (inflammatory bowel disease). All other data are contained in the article and its supplementary information, or are available on request.
