# Supplementary material for: Genome-wide association study identifies 143 loci associated with 25 hydroxyvitamin D concentration
Source: Nat Commun. 2020 Apr 2;11:1647. doi: 10.1038/s41467-020-15421-7 (PMC7118120; doi:10.1038/s41467-020-15421-7)
Supplement: Supplementary file 1 — Supplementary Information [file 41467_2020_15421_MOESM1_ESM.pdf]

SUPPLEMENTARY MATERIAL

Genome-wide association study identifies 143 loci associated with 25 hydroxyvitamin D concentration.

Revez et al

SUPPLEMENTARY METHODS **2**

SUPPLEMENT INTAKE GROUPS 2
PRINCIPAL COMPONENTS 2
SUNLIGHT GWAS SUMMARY STATISTICS 2
IMPUTATION OF THE SUNLIGHT SUMMARY STATISTICS TO 1KGP USING IMPG 2
SAMPLE SIZE BASED META-ANALYSIS 2
UKBR REPLICATION SAMPLE 3

SUPPLEMENTARY NOTES **4**

SUPPLEMENTARY NOTE 1. HERITABILITY AND SNP-BASED HERITABILITY 4
SUPPLEMENTARY NOTE 2. 25OHD AS A BINARY TRAIT 5
SUPPLEMENTARY NOTE 3. SUNLIGHT AND UKB COMPARISONS 6
SUPPLEMENTARY NOTE 4. SUMMARY-DATA-BASED MENDELIAN RANDOMIZATION (SMR) ANALYSES 6
SUPPLEMENTARY NOTE 5. SIMULATION TO INVESTIGATE CONSEQUENCES OF SAMPLE OVERLAP IN GSMR ANALYSES 7

SUPPLEMENTARY FIGURES **8**

SUPPLEMENTARY FIGURE 1. DISTRIBUTION OF 25OHD CONCENTRATIONS IN THE UK BIOBANK SAMPLE INCLUDED IN THIS STUDY (N = 417,580) 8
SUPPLEMENTARY FIGURE 2. HERITABILITY AND SNP-BASED HERITABILITY ESTIMATES OF 25OHD LEVELS ESTIMATED WITH DIFFERENT METHODS. 9
SUPPLEMENTARY FIGURE 3. GREML-LDMS ESTIMATES OF SNP-BASED HERITABILITY 11
SUPPLEMENTARY FIGURE 4. EXAMPLE SMR PLOT 12
SUPPLEMENTARY FIGURE 5. GENETIC CORRELATIONS BETWEEN 25OHD AND SELECTED PHENOTYPES 14
SUPPLEMENTARY FIGURE 6. DISTRIBUTION OF $-\log_{10}$ P-VALUES FROM 20 25OHD VQTLS WITHOUT GWS GXE ASSOCIATION WITH SEASON OF BLOOD DRAW. 15
SUPPLEMENTARY FIGURE 7. UKBR REPLICATION SAMPLE 16
SUPPLEMENTARY FIGURE 8. NUMBER OF COJO GWS LOCI FROM RANDOM DRAWS OF SAMPLES FROM THE UKB SAMPLE 17
SUPPLEMENTARY FIGURE 9. STATISTICAL POWER OF DIFFERENT STUDY DESIGNS TO ESTIMATE GENETIC VARIANCE 18
SUPPLEMENTARY FIGURE 10. EFFECT SIZE ESTIMATES OF GENOME-WIDE SIGNIFICANT LOCI IN A QUANTITATIVE TRAIT AND A SUPER-SCREENED CONTROL DESIGN 19
SUPPLEMENTARY FIGURE 11. FREQUENCY AND EFFECT SIZE OF THE TRAIT-INCREASING ALLELE FOR 142 INDEPENDENT ASSOCIATIONS 20

SUPPLEMENTARY TABLES **21**

SUPPLEMENTARY TABLE 1. 25OHD DISTRIBUTION AND ASSOCIATION WITH POTENTIAL CONFOUNDERS 21
SUPPLEMENTARY TABLE 2. NUMBER OF EFFECTIVE SNPS ESTIMATED FROM SBAYESS AND SBAYESR 23
SUPPLEMENTARY TABLE 3. SBAYESS PARAMETER ESTIMATES 24
SUPPLEMENTARY TABLE 4. RESULTS OF OUT-OF-SAMPLE PREDICTION INTO THE QIMR AND UK BIOBANK REPLICATION (UKBR) DATA SETS 25
SUPPLEMENTARY TABLE 5. MENDELIAN RANDOMIZATION RESULTS BETWEEN 25OHD AND BMI 26
SUPPLEMENTARY TABLE 6. CONDITIONED GWAS RESULTS 27
SUPPLEMENTARY TABLE 7. GSMR RESULTS UNDER THREE SCENARIOS OF SAMPLE OVERLAP 28

SUPPLEMENTARY REFERENCES **29**

SUPPLEMENTARY METHODS

Supplement intake groups

Supplement intake information was obtained from two UKB data fields – 104670 (indicating whether individuals replied to the survey on supplement intake) and 20084 (summarising which supplements were taken). Fields 104670 and 20084 were assessed at 5 instances. Using information from all timepoints, four groups were defined, namely: (1) none (individuals who always reported not taking supplements; N = 90,992), (2) other (individuals who ever reported taking other supplements, but never vitamin D; N = 76,863), (3) vitamin D (individuals who ever reported taking vitamin D, and may also have taken other supplements; N = 11,635), and (4) missing (individuals with no information on supplement intake in any of the five timepoints; N = 238,090).

Principal components

Principal components (PCs) were calculated with UKB genotype calls, which were LD pruned (LD $r^2 < 0.01$) and had long-range LD regions removed by the UKB team. A total of 137,102 variants with MAF > 0.01, HWE *P*-value < 10^{-6} and genotype missingness < 0.05 in unrelated Europeans were used to calculate the PCs with flashPCA[1]. PCs were calculated for unrelated Europeans and projected onto the complete set Europeans.

SUNLIGHT GWAS summary statistics

The SUNLIGHT data were downloaded from https://drive.google.com/drive/folders/0BzYDtCo_doHJRFRKR0ltZHZWZjQ (file 25HydroxyVitaminD_QC.METAANALYSIS1.txt). In total, 79,366 individuals from 31 GWAS cohorts of European descent in Europe, Canada and USA were included in that study. The SUNLIGHT consortium performed genome-wide analyses within each cohort using a uniform analysis plan. Specifically, additive genetic models were fitted using linear regression on natural-log transformed 25OHD. Month of sample collection, sex, age, body mass index, and principal components (PCs) were included as covariates. Then, a fixed-effect inverse variance weighted meta-analysis was conducted using the METAL[2] software package, with control for the population structure. SNPs with a MAF ≤ 0.05, imputation info score ≤ 0.8, HWE ≤ 1×10^{-6}, and less than two studies or 10,000 individuals contributing to each reported SNP association were removed. After quality control, the SUNLIGHT consortium made summary statistics on 2,579,297 SNPs available for download.

Imputation of the SUNLIGHT summary statistics to 1KGP using ImpG

Since individual-level genotypes are not available from the SUNLIGHT consortium, we imputed the SUNLIGHT summary statistics to 1000 Genome Project (1KGP) using ImpG[3]. The haplotype reference panel files (EUR) and SNP mapping files were obtained from 1KGP phase 1 (release v3). Since information on allele frequency was not available in the SUNLIGHT summary statistics, we imputed the allele frequency info using the 1000G imputed ARIC data. We removed SNPs that had mismatched alleles in the two cohorts. After imputation, we further removed SNPs with imputation accuracy metric (r2pred score) < 0.6, and were left with 7,667,712 SNPs. We extracted 6,912,294 SNPs that were available both in the imputed SUNLIGHT and the UKB summary statistics. We further checked the consistency of allele frequencies and the heterogeneity of effect sizes of the SNPs in both cohorts.

Sample size based meta-analysis

Since the effect size estimates (betas) and their standard errors (se) are not in entirely consistent units across the two cohorts, we adopted a sample size-based approach[2] to perform the meta-analysis. This approach converts the direction of effect and *P*-value reported in each cohort into signed z-scores, which are combined across studies into a weighted sum (based on sample size) for each allele. However, since we have SNPs with very small *P*-values, which are recognized as zero by the METAL[2] software and cannot be converted to z-score, we computed the z-score using the betas

and se, and computed the meta-analysis using the same equations and tests in R as implemented in the METAL package.

Among the 6,912,294 SNPs, 6,165 SNPs showed heterogeneity (HetPVal < 1×10^{-3}). Since many of these SNPs were very significant (758 SNPs with $P < 5 \times 10^{-8}$), we kept them for downstream analysis. 15,154 SNPs reached the GWS threshold ($P < 5 \times 10^{-8}$), and 150 SNPs and 166 SNPs were identified as independent signals by the GCTA-COJO and LD clumping analysis.

UKBR replication sample

From the UKB sample we identified 5,402 samples (**Supplementary Figure 7**, green dots) not included in our analysis but who had values of PC1 (PC1 < 0.003) just outside the boundary used for White European (**Supplementary Figure 7**, blue dots). From these we identified 2,384 individuals not related to each other or to anyone in the main analysis (i.e. all GRM off-diagonals <|0.05|. From these we randomly selected 1,632 individuals to match the size of the QIMR sample.

SUPPLEMENTARY NOTES

Supplementary Note 1. Heritability and SNP-based heritability

Our UKB sample included a set of 58,738 individuals related with coefficient of relationship (*r*) > 0.2 to at least one other person in the set ("all relatives"), from whom we estimate the heritability of 25OHD to be 0.32 (s.e. = 0.01) (**Supplementary Figure 2**, **Supplementary Data 1**). Among these, a set of 1st degree relatives (pairs with 0.4 < *r* < 0.6; N = 44,783) and set of 2nd degree relatives (0.2 < *r* < 0.3; N = 15,771) were identified. Heritability estimated from all relatives was 0.32 (s.e. = 0.01). Consistent with some confounding of genetic and shared environment factors between closer family relatives, the heritability estimated from 1st degree relatives (0.33, s.e. = 0.01) was higher than that estimated from 2nd degree relatives alone (0.29, s.e. = 0.04), but the difference was not significant (P = 0.33; Z-test difference between two estimates, H0:difference = 0)

The SNP-based heritability estimate (0.13, 95% CI 0.12-0.14) is higher than estimate from the previous published meta-analysis (SUNLIGHT consortium 0.075%, 95% 0.039-0.11), which may reflect reduced noise in a single cohort study (**Supplementary Figure 8**) as previously been demonstrated for height and BMI[4].

We also estimated SNP-based heritability using three summary-data-based methods: SBayesR[5], SBayesS[6], and LDSC[7]. As expected[8], estimates from GWAS summary statistics were slightly lower than GREML estimates (**Supplementary Figure 2**, **Supplementary Data 1**). SBayesR, a Bayesian method that models SNP effects based on a mixture of normal distributions and a point mass at zero, gave a SNP-based heritability estimate of 0.13 (s.e. = 1.4×10^{-3}). SBayesS is another Bayesian method to estimate SNP-based heritability, which assumes a zero-normal mixture distribution for the SNP effect, with effect size-MAF relationship incorporated as a free parameter (S). The genetic architecture described by the SBayesS π estimate is 0.8% which implies ~9,000 of common SNPs affect variation in 25OHD, and *S* parameter of -0.78 (which represents the relationship between MAF and effect size, and which is zero under a neutral model) which shows evidence of negative selection. These results can be compared to the average of 44 complex traits which had mean SNP-based heritability of 0.18. Of these, the most polygenic traits (body fat percentage, educational attainment and schizophrenia) had about 5% (~55,000) SNPs with nonzero effects, while the least polygenic traits (prostate cancer, age at menopause and male pattern baldness) were affected by about 0.1-0.3% (1,000-3,000) common SNPs. Across these traits the median *S* parameter was = -0.578. The genetic architecture of vitamin D has previously been described as oligogenic, this remains an appropriate descriptor relative to these other complex traits and acknowledges that the architecture includes some variants of comparatively large effect (maximum ~1.6% for rs1352846, **Supplementary Data 3**).

Heritability estimates obtained with LDSC (0.10, s.e. = 0.01) were comparable, but less accurate (larger standard errors), than those obtained with SBayesS. This is likely due to the more conservative method (Jackknife) that LDSC uses to estimate the variance of the estimate

In summer, mean 25OHD levels were always higher than in winter (58.0 vs 41.2 in $nmol{\cdot}L^{-1}$; 0.4 vs. -0.4 RINT SD) (**Supplementary Table 1**). On the other hand, the standard deviation of 25OHD levels measured in summer was only higher than winter when assessment was made on the observed scale (19.8 vs 19.3 in $nmol{\cdot}L^{-1}$; 0.9 vs. 1 in units of RINT(25OHD) applied to the total sample; **Supplementary Figure 1c**). Regardless of the method used for the analysis, the SNP-based heritability for 25OHD levels assessed in summer was always higher than in winter. The genetic correlation between the seasons is (0.80, s.e.= 0.11). While this is not significantly different from one, there may be genetic heterogeneity in 250HD levels between summer and winter, as investigated in GxE analysis.

Supplementary Note 2. 25OHD as a binary trait

While there is some debate about the clinical threshold for vitamin D deficiency (25 nmol·L^{-1} or 30 nmol·L^{-1} serum concentration)[9,10], we chose the more conservative threshold of 25 nmol·L^{-1}. In the UKB sample 12% of participants are vitamin D deficient by this definition. To investigate a hypothesis of non-linear SNP effects on 25OHD we compared our reported results with analyses treating 25OHD as a binary trait. Here, we used 25OHD residuals after regression on known covariates and labelled those with the lowest 12% of values as deficient. We conducted fastGWA analyses on deficient cases (labelled 0 so that the sign of effect sizes aligned with those from the quantitative trait; N = 50,110) with these combinations of non-deficient controls (labelled 1).

1. **Screened controls:** Top 88% in the distribution (i.e. all non-deficient samples, using all observations; N = 367,470; two groups with unequal sample sizes).
2. **Super-screened controls**: Top 12% (i.e. a subsample of the non-deficient sample with the highest 12% 25OHD concentration, N = 50,110; two groups with equal sample sizes).
3. **Down-sampled controls:** Random 12% that are not in the bottom 12% (N = 50,110; two groups with equal sample sizes).

From theory (see Rmarkdown file available cnsgenomics.com) the comparative power of these analyses is shown in **Supplementary Figure 9**. For a locus that explains 0.015% of the variance we have 99% power to detect with 25OHD as a quantitative trait (N = 417,580), 27% power to detect with the screened control data, 83% to detect with the super-screened controls (despite reduced N), and for comparison 0.03% power for the down-sampled controls. Using the estimated variance explained by each of the 142 autosomal loci, calculated as $\sum_{i=1}^{i=142} 2p_i(1-p_i)\widehat{b_i}^2$ where p_i is the allele frequency estimated in the UK Biobank, and $\widehat{b_i}$ is the SNP effect estimate then the relative power assuming linearity of effects is 1 : 0.64 : 0.36 : 0.21 for quantitative trait: super-screened controls; screened controls: down-sampled screened controls. Although the ratios of relative power are not expected to translate directly to ratios of genome-wide significant results, we note that before clumping (which doesn't account for different loci having different LD etc.) we identify 18,337 GWS loci for the quantitative trait and the ratios were 1 : 0.65 : 0.23 : 0.13. After application of COJO to identify independent loci the ratios are: 1 : 0.63 : 0.26 : 0.11 (reflecting 142 : 90 : 37 : 15 COJO SNPs).

Comparing the best powered analysis of the quantitative trait vs. the super-screened binary trait we find a linear relationship in effect size estimates (**Supplementary Figure 10**). These results follow expectation assuming that the binary trait is truncated from a normal distribution.

We note that if only 25OHD deficiency is associated with adverse outcomes, then this finding has important public health implications – interventions designed to increase the 25OHD concentration in the non-deficient population are unlikely to prevent adverse outcomes associated with vitamin D deficiency[11]. Finally, while common variants can influence 25OHD concentrations, it is important to acknowledge that rare variants that abolish key vitamin D-related functions (i.e. homozygous mutations that severely disrupt the function of key enzymes or of the vitamin D receptor) are associated with clinically prominent adverse phenotypes[12] (e.g. bone defects related to impaired calcification). Although a threshold effect is likely to be present, where profoundly lowered vitamin D levels do increase risk of fracture, our mendelian randomization results strongly suggest that increasing levels of vitamin D in the (non-deficient) general population is unlikely to decrease risk of fracture. As the genetic architecture of the broad range of vitamin D-related phenotypes is improved, issues related to potential threshold effects (e.g. disease-specific thresholds for clinical deficiency) should be should be re-examined.

Supplementary Note 3. SUNLIGHT and UKB comparisons

Summary statistics from the SUNLIGHT consortium[13] were available for 2,579,297 SNPs and the genetic correlation estimate with UKB results was not significantly different from 1 ($\hat{r}_g$ = 0.92, s.e. = 0.06). Meta-analysis with our UKB GWAS results after imputation[3,14] of the SUNLIGHT summary statistics (**Supplementary Methods**) (6,912,294 overlapping SNPs) identified 15,154 GWS variants, 150 GCTA-COJO independent SNPs (**Supplementary Methods, Supplementary Data 2**). Of these, 91 were within 1-Mb regions also identified in the UKB alone as GWS. Given that the meta-analysis only increased the total number of significant loci by seven, and given our preference not to include BMI as a covariate, we continued with the UKB-only results for our downstream analyses. Moreover, the only SNP that was significant in the meta-analysis but showed little evidence of association in the UKB (P = 0.17) alone was the known BMI-associated SNP rs1558902, which may be indicative of the ascertainment criteria of some of the cohorts contributing to the SUNLIGHT consortium. It is notable, that random draws of ~80K people from the UKB, the same size approximately as from the SUNLIGHT consortium meta-analysis, identified ~20 independent COJO GWS loci (**Supplementary Figure 8a**). This compares to 14 COJO SUNLIGHT consortium loci, but this increased to 25 when the SUNLIGHT consortium summary statistics were imputed from 2.6M to 7.7M SNPs. Here, we find an approximately linear relationship between sample size and GWS discovery of 3.7 loci/10K people (**Supplementary Figure 8b**).

Supplementary Note 4. Summary-data-based Mendelian randomization (SMR) analyses

To identify 25OHD SNP associations with statistical evidence consistent with a causal/pleiotropic association via gene expression we used summary-data-based Mendelian randomization (SMR)[15] using the 15,504 gene probes with significant cis-eQTLs identified from whole blood eQTLGen data[16]. After Bonferroni correction, we found 112 significantly-associated gene expression probes (P_{SMR} < 3.2 x10^{-6}, i.e., 0.05 / m, with m = 15,504, being the total number of probes tested in SMR analysis; (**Supplementary Data 9;** full details of the SMR analyses can be found on https://cnsgenomics.com). These genes were in 30 broad regions on 16 chromosomes (4 regions on chromosomes 1 and 4; 3 regions on chromosome 11; 2 regions on chromosomes 2, 3, 14, and 19; and 1 region on chromosomes 6, 7, 8, 9, 12, 15, 17 and 20). Genes identified in these analyses included those that encode proteins of interest to vitamin D-related pathways, such as *CYP2R1*, *DHCR7*, *NADSYN1*, *DGAT2* and *HSD17B11*. SMR also confirmed genes of interest discussed for the COJO analyses including *SDR42E1*, *DGAT2* and *POU2F3*. Of the 112 loci identified by SMR, 24 passed the HEIDI test (P_{HEIDI} > 0.05) and have the strongest statistical evidence for a causal role for vitamin D levels (the HEIDI test conservatively excludes results where the gene expression colocalization may be more complex than via the same set of causal SNPs). Of interest, SMR identified a *CYP24A1* intron eQTL variant (rs912505) - this enzyme catalyses additional hydroxylation of 1,25OHD, which leads to the degradation of the active form on vitamin D. SMR also identified eQTL variants related to the expression of (a) *AKR1A1;* a member of the aldo-keto reductase family 1, (b) two hydroxysteroid-related enzymes; *HSD17B13* and *HSD3B7,* and (c) *HAL*; an enzyme expressed in the skin that generates UVB-absorbing molecules.

We then used SMR to explore eQTLs in other relevant tissues (sun-exposed and non-sun-exposed skin, liver, and sixteen brain regions) using data from the GTExV7[16], PsychENCODE[17] and foetal brain tissue[18] (**Supplementary Data 9)**. Of interest, we found significant SMR associations in many tissues for *RP11-660L16.2I*, which is an antisense non-coding RNA located in the bi-directional promoter between *DHCR7* and *NADSYN1*[19]. For both sun-exposed and non-sun-exposed skin, one of the top eQTL variants selected by SMR was rs3819817, within *HAL*. Overall, the SMR-related findings add weight to the hypothesis that these particular eQTL variants may be causally related to 25OHD

concentrations. Plots for all significant SMR results can be found on https://cnsgenomics.com), with an example in **Supplementary Figure 4**.

Supplementary Note 5. Simulation to investigate consequences of sample overlap in GSMR analyses

We had previously shown that Summary-data-based Mendelian randomization (SMR) test statistics are not inflated in the presence of complete sample overlap even when the genetic effects are large[20]. Here, we investigate the consequences of sample overlap for GSMR analysis.

We performed simulations to investigate whether the GSMR estimate of b_{xy} would be biased due to overlapping samples, under three scenarios:

1. Null model: no causal relationship between exposure *x* and outcome *y* ($b_{xy} = 0$) without horizontal pleiotropy;
2. Null model with pleiotropy: No causal relationship, between exposure *x* and outcome *y* ($b_{xy} = 0$), but in the context of horizontal pleiotropy model (i.e. shared causal variants between *x* and outcome *y*).
3. Causal model without pleiotropy: As 1) but with a true causal relationship between exposure x and outcome y.

<u>1. Null model:</u> The simulation was conducted in the imputed UKB data with 1,166,853 HapMap phase 3 SNPs (MAF ≥ 0.01) and 100,000 unrelated individuals of European ancestry. We randomly selected 300 SNPs (z_x) from odd chromosomes as causal variants of exposure (x). The exposure was simulated using the regression model, $x = z_x b_{zx} + e_{zx}$, where b_{zx} is effect of z on x, $b_{zx} \sim N(0, \frac{1}{2f(1-f)})$ with f being allele frequency. The proportion of variance in x captured by z (R^2_{zx}) is 0.2. $e_{zx} \sim N(0, \mathrm{var}(z_x b_{zx})(\frac{1}{R^2_{zx}} - 1))$. We then randomly sampled 300 SNPs (z_y) from even chromosomes as causal variants of outcome (y), using the same method to simulate y with proportion of variance in y explained by z (R^2_{zy}) being 0.2. We performed GWAS analyses to estimate b_{zx} and b_{zy} (effect of z on y). Simulating phenotypes and GWASes were conducted in the same set of individuals. We randomly sampled another 20,000 unrelated individuals from UKB as LD reference sample for GSMR analysis. The simulation was replicated 1,000 times.

<u>2. Null model plus pleiotropy:</u> We simulated both x and y using the same method as above, except to simulate horizontal pleiotropy, we randomly sampled one-third of causal variants (i.e. 100 SNPs) of x and y to be shared between the traits.

<u>3) Causal model:</u> We simulated and standardized x as above. y was simulated using the regression model, $y = x b_{xy} + e_{xy}$, where b_{xy} is the causal effect of x on y with R^2_{xy} (proportion of variance in y explained by x) being 0.1 , and $e_{xy} \sim N(0, \mathrm{var}(x b_{xy})(\frac{1}{R^2_{xy}} - 1))$. y was then standardised. Given the standardisation the value of b_{xy} used in the simulation is arbitrary, but because both x and y were standardised, $\mathrm{E}(\hat{b}_{xy}) = \sqrt{R^2_{xy}} = 0.3162$.

Results over 1,000 replicates are presented in **Supplementary Table 7**. Estimates of b_{xy} do not suffer bias from overlapping samples.

NB: The GSMR *P*-value distribution deviates from uniform under pleiotropy models. As demonstrated above, $\hat{b}_{xy}$ was not biased in either model. If the top-associated SNPs include pleiotropic SNP then the theoretical var($\hat{b}_{xy}$) (mean of var($\hat{b}_{xy}$) estimated by GSMR) would be slightly smaller than empirical var($\hat{b}_{xy}$). Therefore, there is an inflation of small *P*-values is expected under the pleiotropy model.

SUPPLEMENTARY FIGURES

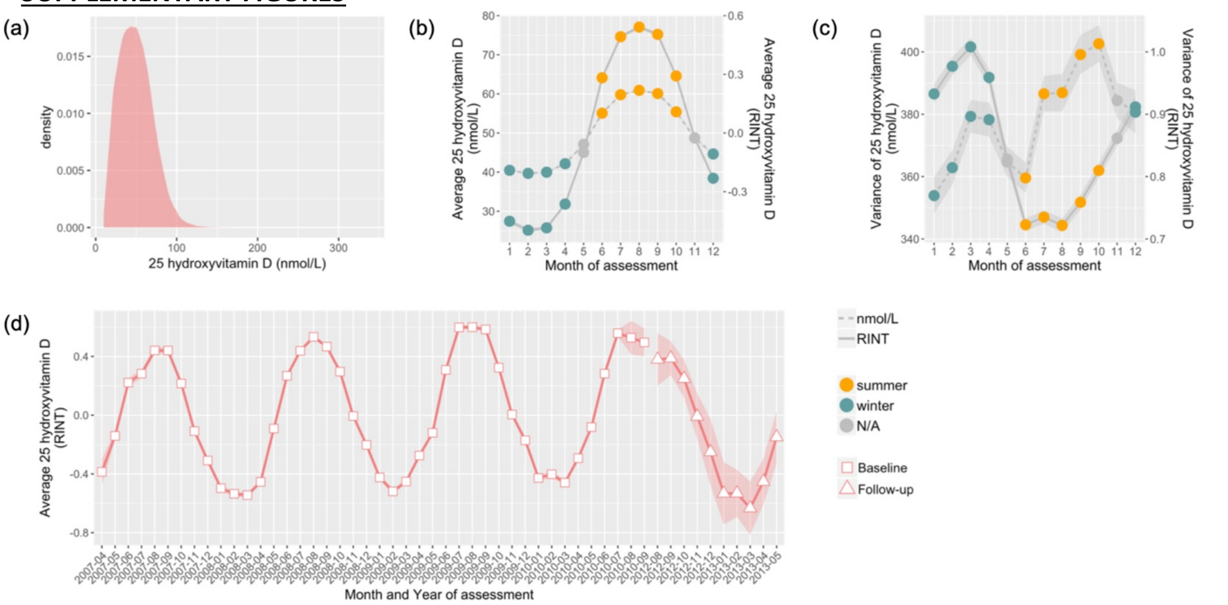

Supplementary Figure 1. Distribution of 25OHD concentrations in the UK Biobank sample included in this study (N = 417,580)

(a) Density distribution of 25 hydroxyvitamin D (25OHD) concentrations (nmol·L^{-1}). The right tail of the distribution had very low counts and is thus obscured by the grid lines. **(b)** Mean monthly 250HD concentrations before and after rank-based inverse-normal transformation (RINT). 95% confidence intervals are shown, but are obscured by the width of the line joining the monthly values. **(c)** Variance between individuals of monthly 25OHD before and after RINT. The lower variance in summer compared to winter is expected under a log transformation (because $\mathrm{var}(\log x) \approx \frac{\mathrm{var}(x)}{\mathrm{mean}(x)^2}$), and also observed under RINT. **(b-c)** Yellow and green dots represent months defined as summer and winter months, respectively. Grey dots represent months that were not included in season-stratified analyses. Shades represent 95% confidence intervals. Dashed and solid lines represent 25OHD before and after RINT, respectively. N_{Jan} = 28,604, N_{Feb} = 33,086, N_{Mar} = 40,971, N_{Apr} = 36,743, N_{May} = 43,191, N_{Jun} = 42,164, N_{Jul}=35,648, N_{Aug} = 31,995, N_{Sep} = 31,286, N_{Oct} = 35,989, N_{Nov} = 34,716, N_{Dec} = 23,187. **(d)** Mean monthly 250HD after RINT across years. Shades represent 95% confidence intervals. Squares represent measurements taken at the baseline visit. Triangles represent measurements collected from participants in first follow-up visit. Months with < 30 observations were not plotted.

A

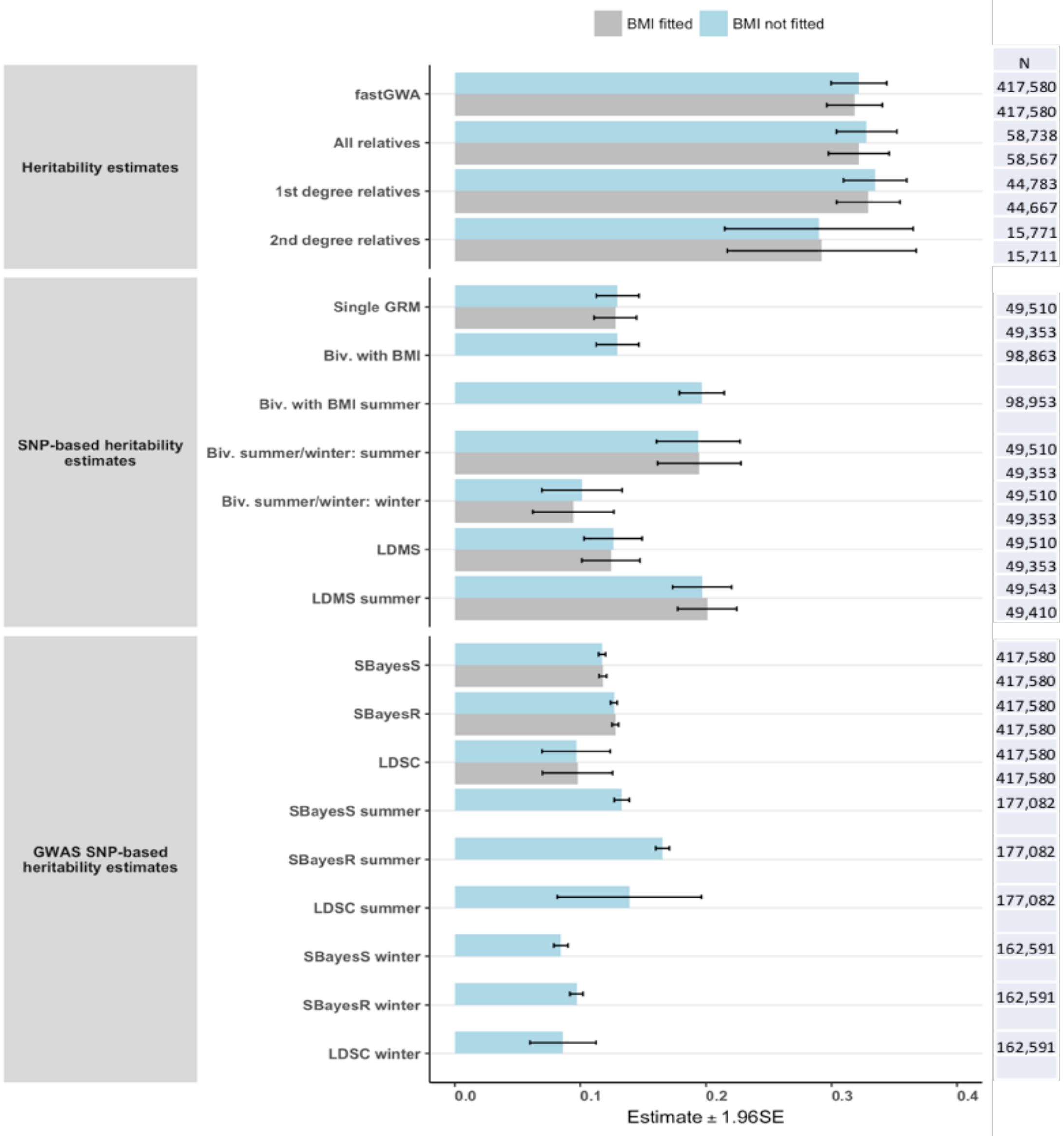

B

	Method	genetic correlation	s.e.
25OHD/BMI	GREML	-0.19	0.03
25OHD/BMI	LDSC	-0.17	0.03
25OHD/BMI (summer)	GREML	-0.18	0.03
Summer/winter	GREML	0.80	0.11
Summer/winter	LDSC	0.89	0.05
Summer/winter (BMI cov)	GREML	0.82	0.11

Supplementary Figure 2. Heritability and SNP-based heritability estimates of 25OHD levels estimated with different methods.

(A) Heritability and SNP-based heritability estimates of 25 hydroxyvitamin D (25OHD) obtained with different methods. Estimates including the words "winter" or "summer" in the label were estimated based on data from the moths Dec – Apr and Jun – Oct, respectively. Most methods were applied with (blue bars) and without (grey bars) BMI fitted as a covariate. Error bars represent the 95% confidence interval of the estimates. The sample size used in each analysis is listed on the right. All estimates are provided in **Supplementary Data 1**. **(B)** Genetic correlation estimates between selected pairs of traits.

Bivariate GREML was conducted between 25OHD and BMI, but only the 25OHD heritability estimates are plotted. Genetic correlation (r_g) estimates and respective standard errors (s.e.) were also obtained from the bivariate GREML analyses. Estimates of genetic correlation between 25OHD and BMI obtained with LDSC regression were -0.17 (s.e. = 0.03) using the UKB data and -0.12 (s.e. = 0.03) using the published BMI meta-analysed results, which excluded the UKB[21]. Bivariate GREML SNP-based (co)heritability analysis estimated a genetic correlation of -0.19 (s.e. = 0.03) and correlation of residual effects of -0.21 (s.e. = 0.05) with BMI.

Abbreviations: GRM, genomic relationship matrix; single GRM analysis contrasts to the linkage disequilibrium minor allele frequency stratified (LDMS) in which the linear mixed model has 10 genetic random effects with 10 GRM constructed from SNPs allocated to 10 bins based on their minor allele frequency and LD score annotation; Biv, bivariate; r_g, genetic correlation.

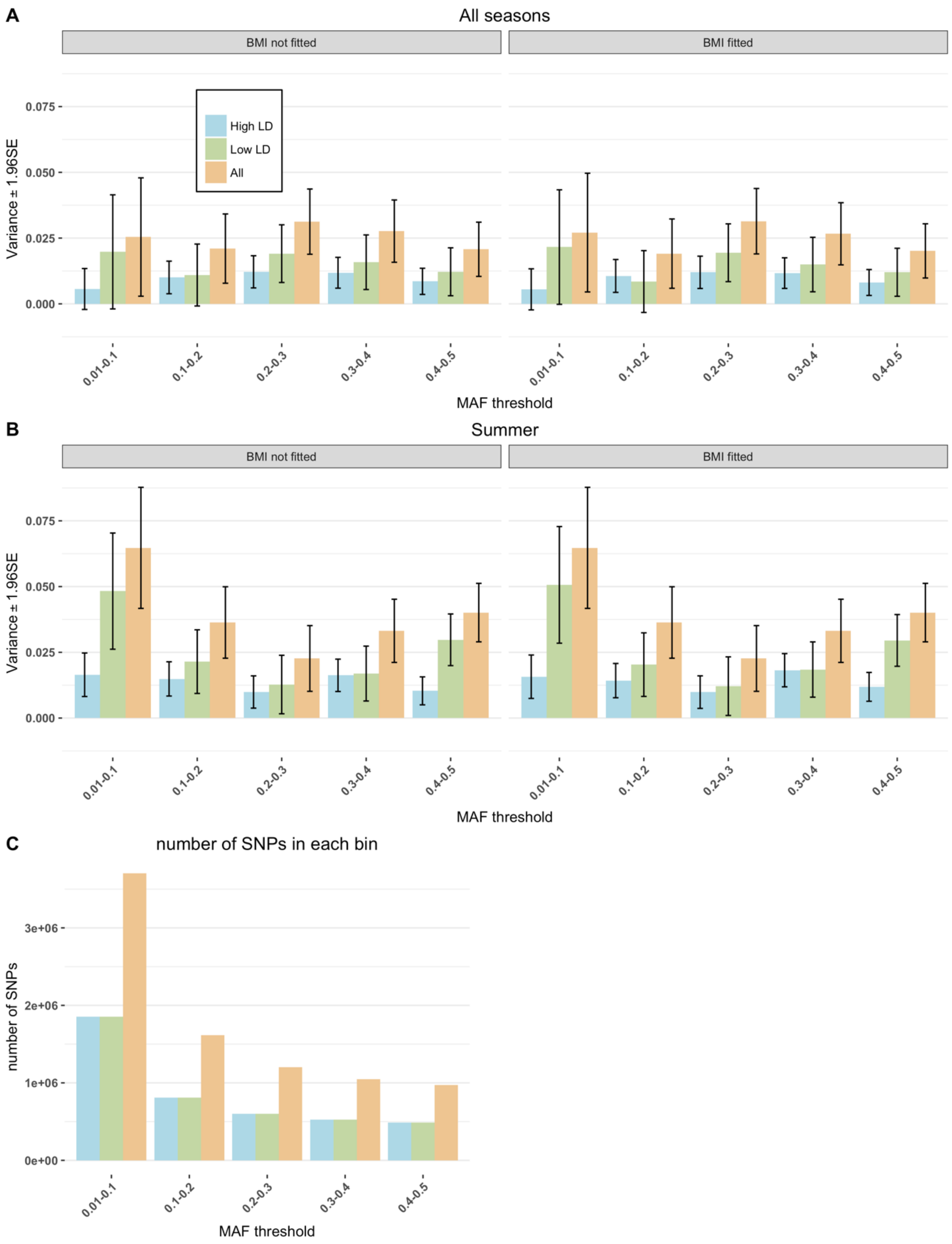

Supplementary Figure 3. GREML-LDMS estimates of SNP-based heritability
(**A-B)** SNP-based heritability estimates stratified by five minor allele frequency (MAF) groups (0.01 – 0.1; 0.1 – 0.2; 0.2 – 0.3; 0.3 – 0.4, 0.4 - 0.5) and two linkage disequilibrium (LD) groups. Blue for high LD, green for low LD, and orange for all SNPs in each MAF bin without LD stratification. Error bars represent the 95% confidence interval of the estimates. **(A)** SNP-based heritability estimates from 25OHD assessments in all months of the year. **(B)** SNP-based heritability estimates from 25OHD assessed in summer months (June – October). (**C)** Number of SNPs in each of the 5 MAF x 2 LD bins.

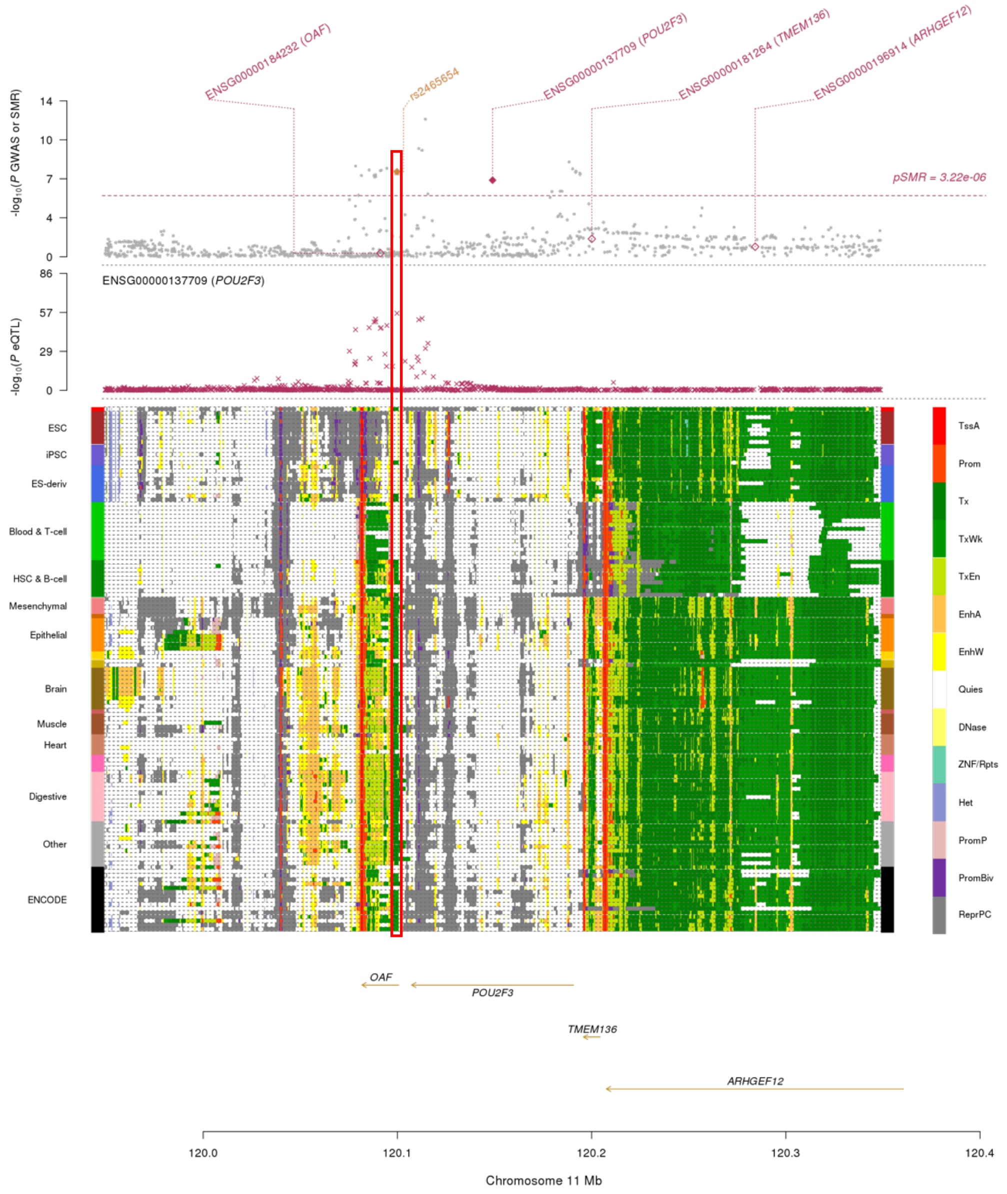

Supplementary Figure 4. Example SMR plot

The above figure shows the results of the SMR+HEIDI analysis that combines the data from GWAS and eQTL studies. Full details of the SMR analyses can be found on https://cnsgenomics.com. Amongst the eQTLs tested, rs2465654 is an eQTL for *POU2F3* in blood and liver (of tissues tested) and generates significant SMR associations to 25OHD. Notably, rs2465654 is not an eQTL in GTEX skin tissue, despite *POU2F3* having massively higher expression in skin compared to other tissues, and where it plays a critical role in keratinocyte proliferation and differentiation. The first track shows -$\log_{10}$(*P*-value) of SNPs (grey dots) from the fastGWA (ref) GWAS of 25OHD. Each red rhombus indicates the -$\log_{10}$(*P*-value) from the SMR tests for associations of gene expression with 25OHD concentration. A solid rhombus represents a probe not rejected by the HEIDI filtering. The yellow rhombus denotes the SNP-25OHD association of the SNP that is the top cis-eQTL (rs2465654). The second track shows -$\log_{10}$(*P*-value) of the SNP association with gene expression probe ENSG00000137709 (tagging *POU2F3*). The third track displays information for 25 chromatin states (indicated by the colours on the right bar) of 127 samples from Roadmap Epigenomics Mapping Consortium (REMC)[22] for different tissues and cell types (indicated by the colours on the left bar),

which communicates potential tissue-specific roles for the SNP. The bottom track shows the genes underlying the genomic region.

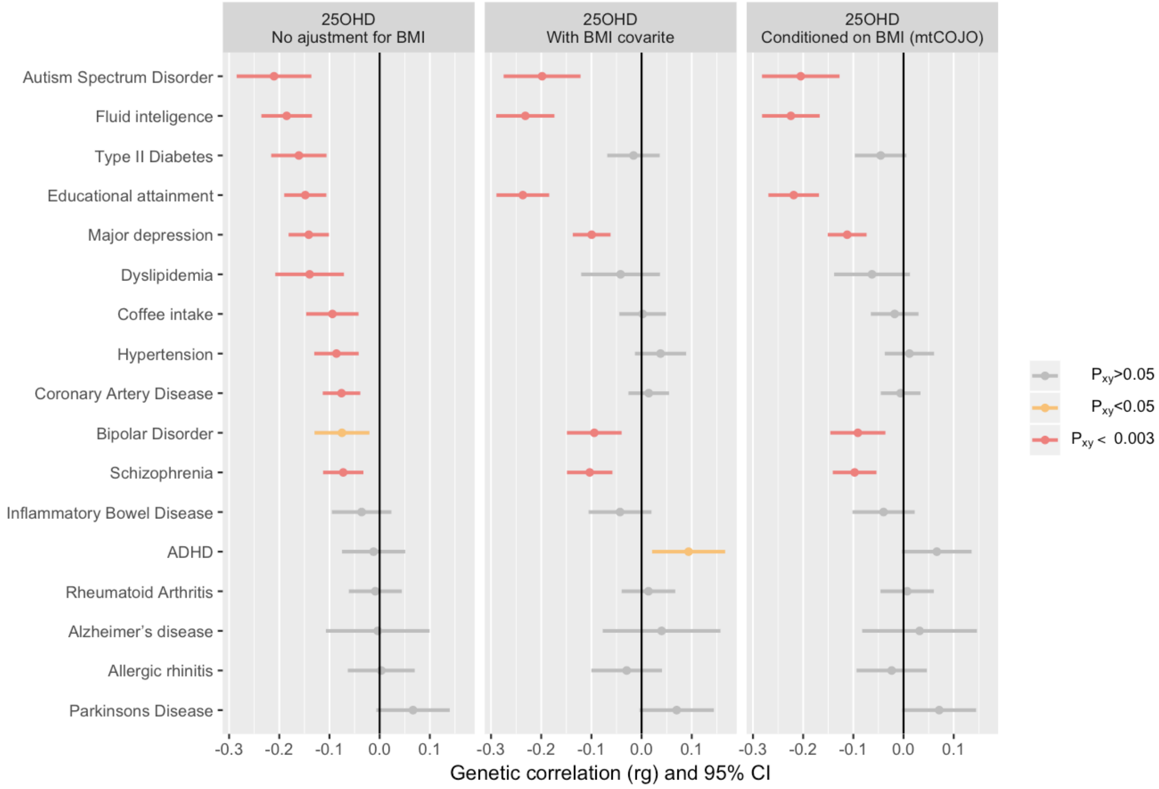

Supplementary Figure 5. Genetic correlations between 25OHD and selected phenotypes

Dots and lines represent the genetic correlation estimates ($\hat{r}_g$) and respective 95% confidence intervals between variants associated with 25 hydroxyvitamin D (25OHD) and selected phenotypes. Genetic correlations were estimated from bivariate LDSC regression[23] using GWAS summary statistics from 25OHD and selected phenotypes. Correlations are shown for 25OHD GWAS analyses that (1) make no adjustment for body mass index (BMI), (2) include BMI as a covariate, or (3) condition on genetic correlates of BMI (via mtCOJO)[24]. Negative genetic correlations indicate the variants associated with increased 25OHD concentration were associated with a smaller value/reduced risk for the phenotypes of interest. Non-significant (correlation $P > 0.05$) $\hat{r}_g$ are presented in grey. Nominally significant (correlation $P < 0.05$) $\hat{r}_g$ are presented in yellow. $\hat{r}_g$ that remained significant after Bonferonni correction for multiple testing (correlation $P < 0.003$) are presented in red. All $\hat{r}_g$ and respective standard errors and *P*-values can be found in tabular form in **Supplementary Data 10**. For additional information about the summary statistic related to these phenotypes, see the original publications as provided in **Supplementary Data 10.**

Min.	1st Qu.	Median	Mean	3rd Qu.	Max.
1.0E-07	0.0001	0.0286	0.1863	0.2335	0.9093

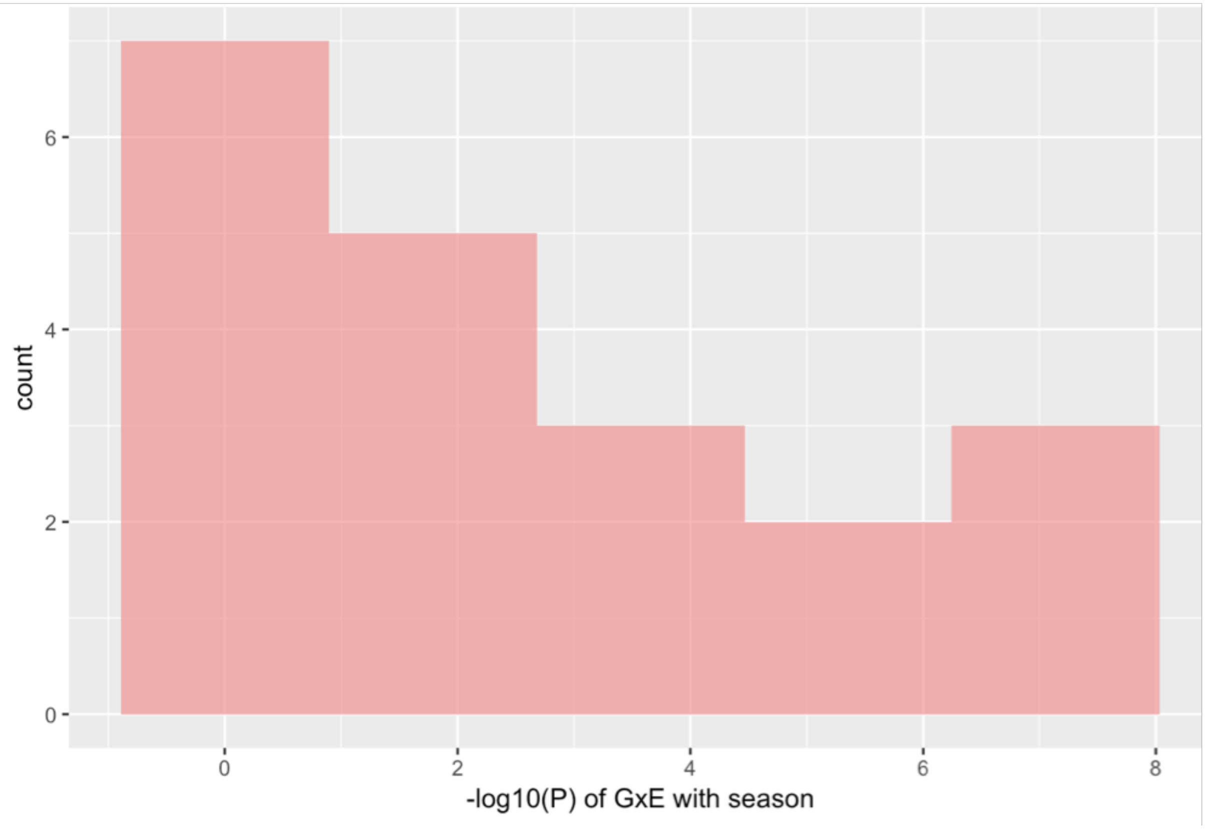

Supplementary Figure 6. Distribution of -log$_{10}$ P-values from 20 25OHD vQTLs without GWS GxE association with season of blood draw.

The median linear regression *P*-value in the GxE analysis is 0.03, which suggests that at least half of these 20 vQTL are unlikely to have SNP*season interaction. GWS: genome-wide significant.

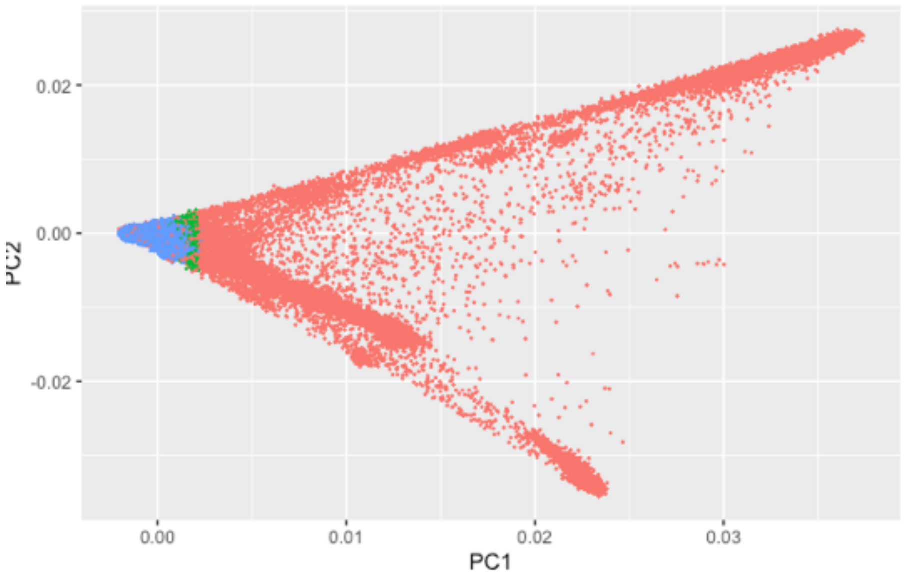

Supplementary Figure 7. UKBR replication sample
UK Biobank (UKB) samples projected into principal components (PC) 1 and 2. Blue dots represent European samples. Red dots represent non-European samples. Green dots represent 5,402 samples just outside the boundary used for White European, from which 1,632 individuals unrelated to each other, or to anyone in the main analysis, were selected as the UKB replication (UKBR) sample.

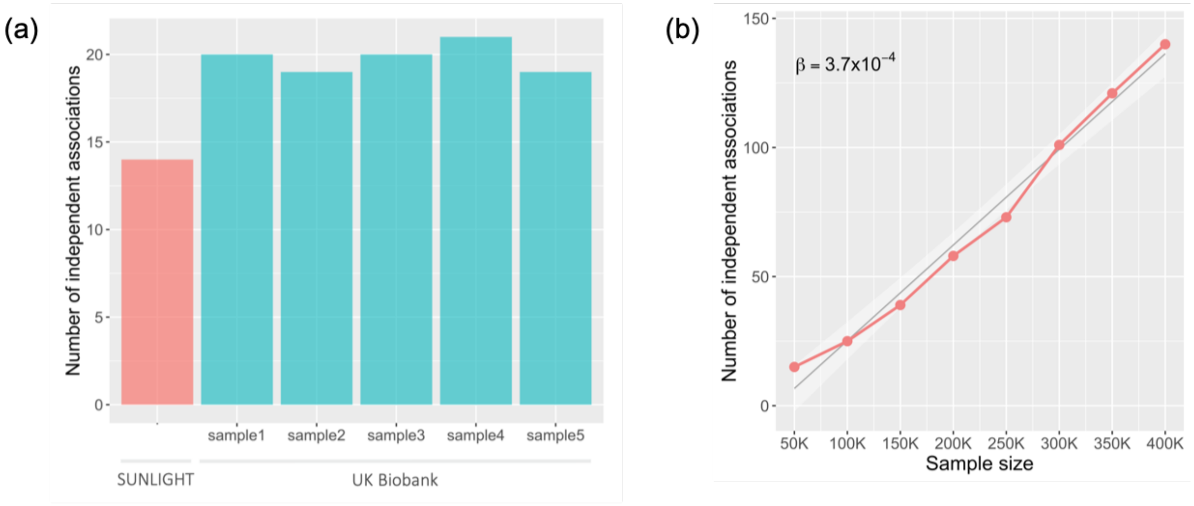

Supplementary Figure 8. Number of COJO GWS loci from random draws of samples from the UKB sample

(a) Five random draws of 80K samples (the same size as the SUNLIGHT consortium) identify ~20 GWS loci. SUNLIGHT identifies 14 COJO GWS loci (which increased to 25 when the SUNLIGHT consortium summary statistics were imputed from 2.6M to 7.7M SNPs). **(b)** There is an approximately linear trend of 3.7 number loci per 10,000 individuals. GWS: genome-wide significant. COJO: conditional and joint analysis (see Methods)

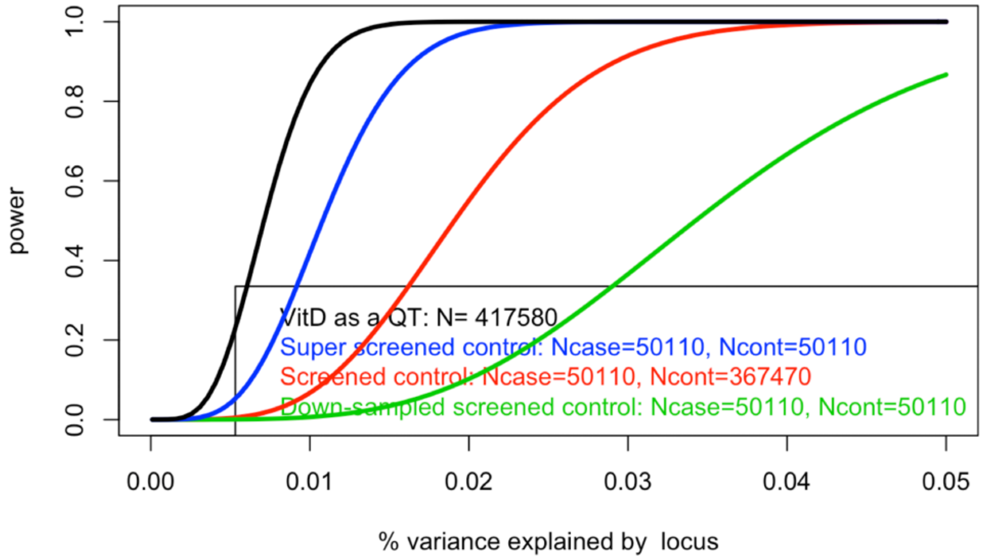

Supplementary Figure 9. Statistical power of different study designs to estimate genetic variance
Statistical power to detect the effect of a locus depending on the percentage of variance it explains (x-axis) and the study design used. Specifically, four study designs are compared, namely 25OHD assessed as a (1) quantitative trait (QT; black line); (2) binary trait with super screened controls (blue line); (3) binary trait with screened controls (red line); and (4) binary trait with down-sampled controls (green line). The super-screened control design uses the same proportion of cases and controls (i.e. top 12% observations are controls and bottom 12% are cases). The screened control design uses the all controls (i.e. top 88% observations as controls and bottom 12% are cases). The down-sampled design uses the same proportion of cases and controls, but controls are randomly sampled from the bottom 88%.

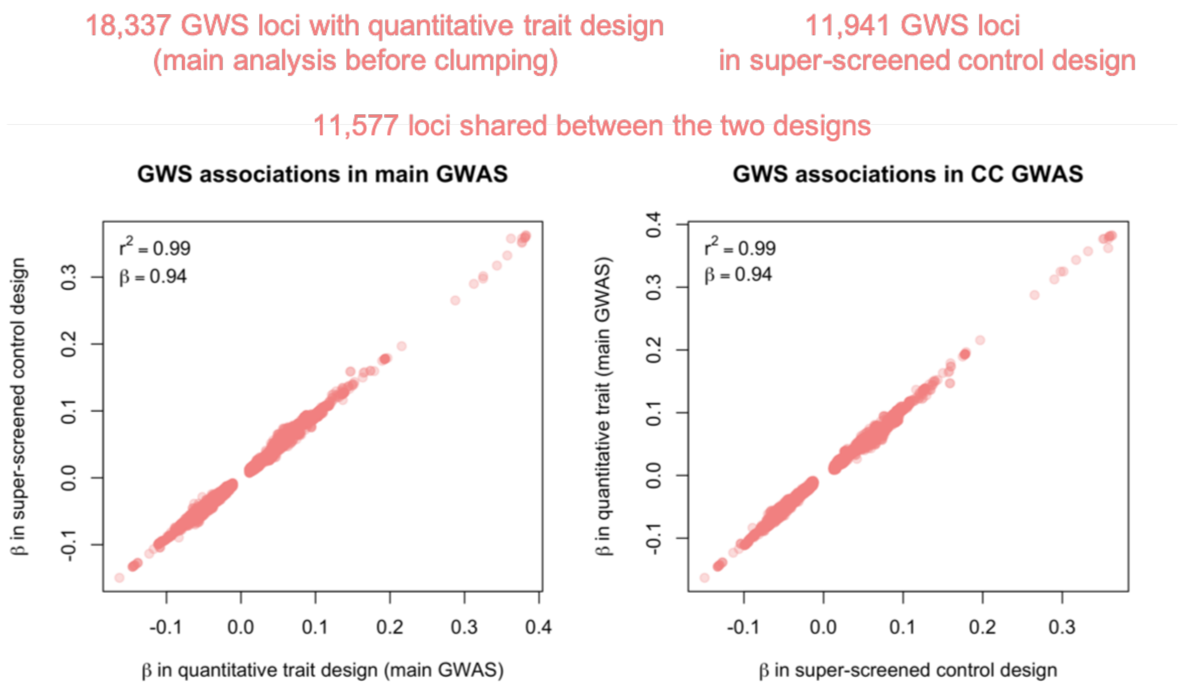

Supplementary Figure 10. Effect size estimates of genome-wide significant loci in a quantitative trait and a super-screened control design

Effect sizes of genome-wide significant (GWS) loci identified with two study designs: 25OHD as a (1) quantitative trait, and (2) super-screened control design (i.e. same proportion of cases and controls, with cases and controls drawn from the bottom and top of the distribution, respectively).

NB These graphs show more positive associations than negative but this reflects, in part, the chance choice of alleles (**Supplementary Figure 11**). Also, we note that the beta values are on different scales for the quantitative and super-screened controls. As expected from theory the correlation in effect size between super-screened controls and the quantitative trait is higher than between screened controls and the quantitative trait.

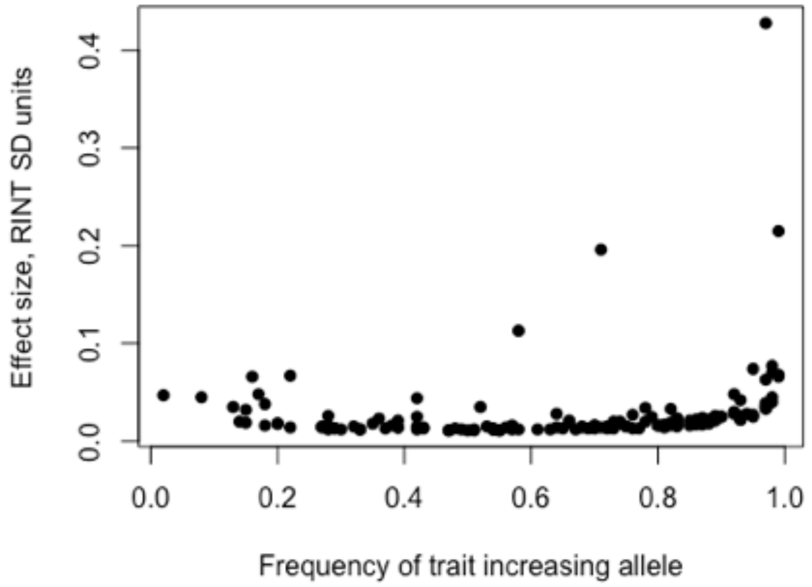

Supplementary Figure 11. Frequency and effect size of the trait-increasing allele for 142 independent associations
For the 142 autosomal loci (identified with COJO) we compared the allele frequency of the trait increasing allele (x-axis) with its effect size (y-axis).

SUPPLEMENTARY TABLES

Supplementary Table 1. 25OHD distribution and association with potential confounders

Associations with potential confounders were tested with linear regression (25OHD dependent variable for continuous and categorical independent variables). RINT and log transformations presented below were applied to the whole sample (i.e. before stratifying in summer and winter cohorts). For association analyses, RINT was re-applied after stratifying the whole cohort by season. Abbreviations: beta, effect size estimate; IQR, interquartile range; N/A, not presented for categorical covariates; r^2, variance explained; RINT, Rank Inverse Normal Transformation; SD, standard deviation.

	Distribution								
	25OHD (nmol·L^{-1})			RINT(25OHD)			log(25OHD)		
	All	Summer	Winter	All	Summer	Winter	All	Summer	Winter
N	417,580	177,082	162,591	417,580	177,082	162,591	417,580	177,082	162,591
Mean	49.6	58.0	41.2	0.000	0.413	-0.418	3.8	4.0	3.6
Median	47.9	56.9	37.7	0.000	0.395	-0.465	3.9	4.0	3.6
SD	21.0	19.8	19.3	1.000	0.873	0.986	0.5	0.4	0.5
IQR	33.5 - 63.2	44.1 - 70.2	26.2 - 53	-0.674 - 0.674	-0.166 - 0.984	-1.093 - 0.226	3.5 - 4.2	3.8 - 4.3	3.3 - 4.0

	Association with potential confounders								
	25OHD (nmol·L^{-1})			RINT(25OHD)			log(25OHD)		
	beta	r^2	*P*-value	beta	r^2	*P*-value	beta	r^2	*P*-value
Age	0.193	0.006	0.0E+00	0.010	0.006	0.0E+00	0.005	0.007	0.0E+00
Assessment centre	N/A	0.029	0.0E+00	N/A	0.032	0.0E+00	N/A	0.034	0.0E+00
Assessment month	N/A	0.140	0.0E+00	N/A	0.150	0.0E+00	N/A	0.160	0.0E+00
Genotyping batch	N/A	0.001	1.1E-30	N/A	0.001	4.0E-38	N/A	0.001	1.2E-43
BMI	-0.757	0.030	0.0E+00	-0.036	0.030	0.0E+00	-0.016	0.029	0.0E+00
PC1	-36.373	0.001	7.5E-75	-1.997	0.001	1.1E-98	-1.042	0.001	5.1E-126
PC2	-86.409	0.003	4.1E-280	-4.421	0.004	0.0E+00	-2.140	0.004	0.0E+00
PC3	-61.373	0.001	9.7E-78	-3.038	0.001	1.1E-83	-1.404	0.001	3.5E-84

PC4	-20.649	7.7E-05	1.5E-08	-1.195	1.1E-04	6.0E-12	-0.633	1.5E-04	2.6E-15
PC5	3.772	2.4E-06	0.3170	0.127	1.2E-06	4.8E-01	0.048	8.0E-07	5.6E-01
PC6	2.771	1.3E-06	0.4630	0.180	2.4E-06	3.2E-01	0.108	4.0E-06	1.9E-01
PC7	5.698	5.4E-06	0.1320	0.283	5.9E-06	1.2E-01	0.137	6.5E-06	1.0E-01
PC8	3.797	2.4E-06	0.3200	0.138	1.4E-06	4.5E-01	0.046	7.2E-07	5.8E-01
PC9	-0.100	1.6E-09	0.9790	0.038	1.0E-07	8.4E-01	0.030	3.1E-07	7.2E-01
PC10	4.793	3.6E-06	0.2190	0.319	7.1E-06	8.6E-02	0.177	1.0E-05	3.9E-02
Sex	0.064	2.3E-06	0.3250	0.003	2.3E-06	3.2E-01	0.001	2.4E-06	3.2E-01
Supplement intake	N/A	0.032	0.0E+00	N/A	0.033	0.0E+00	N/A	0.034	0.0E+00

Supplementary Table 2. Number of effective SNPs estimated from SbayesS and SBayesR
In SBayesR, SNPs with small, medium and large effect sizes are the SNPs whose effect size follows a normal distribution with mean zero and variance of 0.01, 0.1, and 1 times σ^2, respectively, where σ^2 is common to all SNPs. Abbreviations: NnzSnp, number of SNPs with zero effect.

	Group of SNPs	all	summer	winter	all with BMI
SBayesR	SNPs with no effect	1,070,539.25	1,082,051.13	1,079,923.88	1,069,291.75
	SNPs with small effect	23,818.82	12,398.32	14,645.28	24,987.12
	SNPs with medium effect	219.83	137.19	47.29	286.78
	SNPs with large effect	72	63.36	33.53	84.28
	All SNPs with effect	24,110.64	12,598.88	14,726.10	25,358.17
SBayesS	NnzSnp	9,070.66	1,996.83	3,949.69	8,434.85

Supplementary Table 3. SBayesS parameter estimates

Abbreviations: hsq, GWAS SNP-based heritability estimate; NnzSnp, number of SNPs with zero effect; Pi, parameter indicating polygenicity; S, SBayesS *S* parameter, which describes the relationship between the effect size and minor allele frequency; SD, standard deviation.

	all		summer		winter		all with BMI	
	Mean	SD	Mean	SD	Mean	SD	Mean	SD
Pi	0.0083	0.0004	0.0018	0.0002	0.0036	0.0004	0.0077	0.0003
NnzSnp	9,070.66	372.92	1,996.83	194.72	3,949.69	396.52	8,434.85	355.48
S	-0.78	0.04	-0.82	0.08	-0.77	0.07	-0.79	0.04
hsq	0.12	0.00	0.13	0.00	0.08	0.00	0.12	0.00

Supplementary Table 4. Results of out-of-sample prediction into the QIMR and UK biobank replication (UKBR) data sets

beta: standard deviation (SD) units in rank inverse normal transformed residuals of 25OHD (after fitting covariates) per SD unit of standardised polygenic risk scores. Abbreviations: beta, effect size estimate; r^2, variance explained; s.e., standard error.

	QIMR (N=1,632)				UKBR (N=1,632)			
Threshold	r^2	*P*-value	beta	s.e.	r^2	*P*-value	beta	s.e.
p<5x10-8	0.0749	3.7E-31	0.24	0.020	0.0358	1.3E-14	0.19	0.024
p<1x10-5	0.0729	2.6E-30	0.24	0.021	0.0428	3.2E-17	0.21	0.024
p<0.001	0.0650	4.7E-27	0.23	0.021	0.0339	6.3E-14	0.18	0.024
p<0.01	0.0574	6.8E-24	0.21	0.021	0.0167	1.7E-07	0.13	0.025
p<0.05	0.0452	7.6E-19	0.19	0.021	0.0119	1.0E-05	0.11	0.025
p<0.1	0.0402	1.0E-16	0.18	0.021	0.0110	2.1E-05	0.10	0.025
p<0.5	0.0378	1.1E-15	0.17	0.021	0.0077	3.7E-04	0.09	0.025
p<1	0.0390	3.5E-16	0.17	0.021	0.0076	4.3E-04	0.09	0.025

Prediction with 143 COJO SNPs, and SBayesS and SBayesR results								
Method	R2	Pval	beta	s.e	R2	Pval	beta	s.e
COJO	0.105	1.44E-43	0.288	0.020	0.0355	1.6E-14	0.19	0.024
SBayesS	0.086	1.12E-34	0.255	0.020	0.0566	2.0E-22	0.24	0.024
SBayesR	0.096	7.75E-39	0.269	0.020	0.0554	5.7E-22	0.24	0.024

Supplementary Table 5. Mendelian randomization results between 25OHD and BMI
Analyses were conducted using the two sample Mendelian randomisation (2SMR) R package (Hemani et al. 2018). For maximum comparability between methods, we used the same SNP instruments that were selected in the GSMR analyses, using the default in-built GSMR clumping step. Palindromic SNPs were excluded. Abbreviations: beta, effect size estimate; nsnp, number of SNP instruments; s.e., standard error.

exposure	outcome	method	heidi	nsnp	beta	s.e.	*P*-value
BMI	25OHD	MR Egger	noHeidi	1064	-0.115	0.0205	2.4E-08
BMI	25OHD	Weighted median	noHeidi	1064	-0.111	0.0080	7.3E-44
BMI	25OHD	Inverse variance weighted	noHeidi	1064	-0.106	0.0073	6.3E-48
BMI	25OHD	Simple mode	noHeidi	1064	-0.177	0.0347	4.4E-07
BMI	25OHD	Weighted mode	noHeidi	1064	-0.163	0.0281	9.4E-09
BMI	25OHD	MR Egger	withHeidi	994	-0.126	0.0158	3.9E-15
BMI	25OHD	Weighted median	withHeidi	994	-0.118	0.0079	5.3E-50
BMI	25OHD	Inverse variance weighted	withHeidi	994	-0.128	0.0057	2.2E-112
BMI	25OHD	Simple mode	withHeidi	994	-0.177	0.0360	9.9E-07
BMI	25OHD	Weighted mode	withHeidi	994	-0.167	0.0300	3.5E-08
25OHD	BMI	MR Egger	noHeidi	274	0.023	0.0196	0.2480
25OHD	BMI	Weighted median	noHeidi	274	0.001	0.0096	0.8890
25OHD	BMI	Inverse variance weighted	noHeidi	274	-0.014	0.0140	0.3320
25OHD	BMI	Simple mode	noHeidi	274	0.004	0.0208	0.8520
25OHD	BMI	Weighted mode	noHeidi	274	0.004	0.0080	0.6300
25OHD	BMI	MR Egger	withHeidi	208	0.002	0.0086	0.8090
25OHD	BMI	Weighted median	withHeidi	208	0.001	0.0100	0.8930
25OHD	BMI	Inverse variance weighted	withHeidi	208	0.001	0.0061	0.8680
25OHD	BMI	Simple mode	withHeidi	208	0.007	0.0233	0.7760
25OHD	BMI	Weighted mode	withHeidi	208	0.003	0.0078	0.7400

Supplementary Table 6. Conditioned GWAS results

Genome-wide association study (GWAS) results before and after conditioning on the most significant common variant in *CYP2R1* (identified by GCTA-COJO)[25], rs12794714, and the most significant low frequency variant in *PDE3B*/*CYP2R1* (identified by GCTA-COJO), rs116970203. Abbreviations: beta, effect size estimate; bC, conditional effect size estimate; Chr, chromosome; EA, effect allele; EAF, frequency of the effect allele; pC, conditional *P*-value; SNP, single nucleotide polymorphism.

							Conditional on rs12794714		Conditional on rs116970203	
Chr	SNP	Position	EA	EAF	beta	*P*-value	bC	pC	bC	pC
11	rs61883501	13882754	A	0.97	0.002	0.7492	-0.012	0.0373	0.006	0.2683
11	rs116970203	14876718	G	0.97	0.377	0.0E+00	0.415	0.0E+00	NA	NA
11	rs117576073	14912573	G	0.99	0.147	5.9E-62	0.182	1.1E-93	0.159	2.8E-72
11	rs12794714	14913575	G	0.58	0.088	0.0E+00	NA	NA	0.105	0.0E+00
11	rs61891388	66079818	T	0.54	-0.013	4.1E-10	-0.013	4.1E-10	-0.013	4.1E-10
11	rs78168201	70971149	C	0.99	-0.089	5.2E-25	-0.089	5.3E-25	-0.089	5.3E-25
11	rs1660839	71094232	G	0.75	-0.029	6.4E-37	-0.029	6.6E-37	-0.029	6.6E-37
11	rs12803256	71132868	A	0.22	-0.104	0.0E+00	-0.104	0.0E+00	-0.104	0.0E+00
11	rs12798050	71223256	C	0.17	-0.110	0.0E+00	-0.110	0.0E+00	-0.110	0.0E+00
11	rs72997623	75488054	C	0.92	-0.028	1.3E-14	-0.028	1.3E-14	-0.028	1.3E-14
11	rs1149605	76485216	T	0.83	-0.022	1.3E-16	-0.022	1.3E-16	-0.022	1.3E-16
11	rs964184	116648917	G	0.13	-0.043	1.1E-48	-0.043	1.2E-48	-0.043	1.2E-48
11	rs613808	116710968	A	0.28	-0.026	3.4E-32	-0.026	3.5E-32	-0.026	3.5E-32
11	rs2847500	120114421	G	0.88	0.022	4.4E-13	0.022	4.4E-13	0.022	4.4E-13

Supplementary Table 7. GSMR results under three scenarios of sample overlap

We performed simulations to investigate whether the GSMR estimate of b_{xy} would be biased due to overlapping samples, under three scenarios: (1) Null model: no causal relationship between exposure x and outcome y (b_{xy}=0) without horizontal pleiotropy; (2) Null model with pleiotropy: No causal relationship, between exposure x and outcome y (b_{xy}=0), but in the context of horizontal pleiotropy model (i.e. shared causal variants between x and outcome y); and (3) Causal model without pleiotropy: As 1) but with a true causal relationship between exposure x and outcome y. The table presents results over 1,000 replicates.

		$\hat{b}_{xy}$ mean	SE of mean
1	Null : b_{xy} = 0	0.00013	0.00023
2	b_{xy} = 0 & Pleiotropy	-0.00052	0.00033
3	Causal: b_{xy} = 0.3162	0.3161	0.00022

SUPPLEMENTARY REFERENCES

1. Abraham, G. & Inouye, M. Fast principal component analysis of large-scale genome-wide data. *PLoS One* **9**, e93766 (2014).
2. Willer, C.J., Li, Y. & Abecasis, G.R. METAL: fast and efficient meta-analysis of genomewide association scans. *Bioinformatics* **26**, 2190-1 (2010).
3. Pasaniuc, B. *et al.* Fast and accurate imputation of summary statistics enhances evidence of functional enrichment. *Bioinformatics* **30**, 2906-14 (2014).
4. Yengo, L. *et al.* Meta-analysis of genome-wide association studies for height and body mass index in approximately 700000 individuals of European ancestry. *Hum Mol Genet* **27**, 3641-3649 (2018).
5. Zeng, B. *et al.* Comprehensive Multiple eQTL Detection and Its Application to GWAS Interpretation. *Genetics* **212**, 905-918 (2019).
6. Zeng, J. *et al.* Bayesian analysis of GWAS summary data reveals differential signatures of natural selection across human complex traits and functional genomic categories. *bioRxiv*, 752527 (2019).
7. Bulik-Sullivan, B.K. *et al.* LD Score regression distinguishes confounding from polygenicity in genome-wide association studies. *Nat Genet* **47**, 291-5 (2015).
8. Evans, L.M. *et al.* Narrow-sense heritability estimation of complex traits using identity-by-descent information. *Heredity (Edinb)* **121**, 616-630 (2018).
9. Institute of Medicine. *Dietary Reference Intakes for Calcium and Vitamin D*, (National Academies Press, 2010).
10. Holick, M.F. Vitamin D deficiency. *N Engl J Med* **357**, 266-81 (2007).
11. Trajanoska, K. *et al.* Assessment of the genetic and clinical determinants of fracture risk: genome wide association and mendelian randomisation study. *BMJ* **362**, k3225 (2018).
12. Malloy, P.J. & Feldman, D. Genetic disorders and defects in vitamin d action. *Endocrinol Metab Clin North Am* **39**, 333-46, table of contents (2010).
13. Jiang, X. *et al.* Genome-wide association study in 79,366 European-ancestry individuals informs the genetic architecture of 25-hydroxyvitamin D levels. *Nat Commun* **9**, 260 (2018).
14. Genomes Project, C. *et al.* A global reference for human genetic variation. *Nature* **526**, 68-74 (2015).
15. Zhu, Z. *et al.* Integration of summary data from GWAS and eQTL studies predicts complex trait gene targets. *Nat Genet* **48**, 481-7 (2016).
16. Võsa, U. *et al.* Unraveling the polygenic architecture of complex traits using blood eQTL metaanalysis. *bioRxiv*, 447367 (2018).
17. Gandal, M.J. *et al.* Transcriptome-wide isoform-level dysregulation in ASD, schizophrenia, and bipolar disorder. *Science* **362**(2018).
18. O'Brien, H.E. *et al.* Expression quantitative trait loci in the developing human brain and their enrichment in neuropsychiatric disorders. *Genome Biology* **19**, 194 (2018).
19. Odhams, C.A. *et al.* Mapping eQTLs with RNA-seq reveals novel susceptibility genes, non-coding RNAs and alternative-splicing events in systemic lupus erythematosus. *Hum Mol Genet* **26**, 1003-1017 (2017).
20. Wu, Y. *et al.* Integrative analysis of omics summary data reveals putative mechanisms underlying complex traits. *Nat Commun* **9**, 918 (2018).
21. Locke, A.E. *et al.* Genetic studies of body mass index yield new insights for obesity biology. *Nature* **518**, 197-206 (2015).
22. Bernstein, B.E. *et al.* The NIH Roadmap Epigenomics Mapping Consortium. *Nat Biotechnol* **28**, 1045-8 (2010).
23. Bulik-Sullivan, B. *et al.* An atlas of genetic correlations across human diseases and traits. *Nat Genet* **47**, 1236-41 (2015).

24. Zhu, Z. *et al.* Causal associations between risk factors and common diseases inferred from GWAS summary data. *Nat Commun* **9**, 224 (2018).
25. Yang, J. *et al.* Conditional and joint multiple-SNP analysis of GWAS summary statistics identifies additional variants influencing complex traits. *Nat Genet* **44**, 369-75, S1-3 (2012).
