# Supplementary material for: Genome-wide association study identifies 143 loci associated with 25 hydroxyvitamin D concentration
Source: Nat Commun. 2020 Apr 2;11:1647. doi: 10.1038/s41467-020-15421-7 (PMC7118120; doi:10.1038/s41467-020-15421-7)
Supplement: Supplementary file 2 — Description of Additional Supplementary Files [file 41467_2020_15421_MOESM2_ESM.pdf]

Title: Supplementary Data 1. Heritability and SNP-based heritability estimates

Description: bigK, heritability from close relationships (see https://cnsgenomics.com/software/gcta/#GREMLinfamilydata); Biv., bivariate; BMI, body mass index; GRM, genetic relationship matrix; Heritability, narrow-sense heritability, i.e. proportion of the phenotypic variance that is explained by genetic factors ($V_G \cdot V_P^{-1}$). NB, when two GRM are fitted $h^2 = (V_G bigK + V_{G\_GRM}) \cdot V_P^{-1}$; N, sample size; r_g, genetic correlation estimate; SNPs single nucleotide polymorphisms; s.e., standard error; V_G, genetic variance; V_P, phenotypic variance.

Title: Supplementary Data 2. Summary of genome-wide associations and comparison of GWAS results generated with three levels of correction for BMI

Description: Three sets of GWAS results were generated with varying levels of correction for BMI, namely: (1) no correction, (2) including BMI as covariate, and (3) conditioning on BMI, with mtCOJO (Zhu et al. 2018). GWAS were conducted using the linear mixed model implemented in fastGWA (Jiang et al. 2019). Below we present the number of genome-wide significant (GWS) associations and the number of independent associations identified with conditional and joint analysis (COJO) (Yang et al. 2012) in each of the analysis. Results are available for less SNPs in the BMI-conditioned analysis because some SNPs were not tested in the BMI GWAS (Xue et al. 2018) used to condition the 25OHD GWAS results. Abbeviations: GWS, genome-wide significant; N, sample size.

Title: Supplementary Data 3. 143 independent associations identified with GCTA-COJO (conditional and joint) analysis applied to GWAS summary statistics (no adjustment for BMI) and the results for these loci from replication analyses

Description: Presented are the (1) UK Biobank (UKB) GWAS summary statistics (yellow header), (2) QIMR replication analysis summary statistics (green header), (3) UKB replication analysis summary statistics (blue header), (4) imputed SUNLIGHT summary statistics (light yellow header), and (5) summary statistics from meta-analysis of UKB and SUNLIGHT consortium. The UKB GWAS summary statistics are presented before and after conditional and joint (COJO) analysis (Yang et al. 2012) and also after conditioning on BMI (using mtCOJO(Zhu et al. 2018)). Some variants do not have BMI-conditioned results because no results were available in the BMI GWAS (Xue et al. 2018) used to condition the 25OHD association results. QIMR replication analysis summary statistics were generated from 1,632 individuals. UKB replication analysis summary statistics were generated from 1,632 unrelated individuals that were also unrelated to those in the main UKB GWAS. Meta-analysis of UKB and SUNLIGHT consortium was conducted with results from the published SUNLIGHT consortium meta-analysis and log-transformed 25OHD UKB GWAS with BMI as covariate. Chromosome X is coded as chromosome 23.

Columns are: SNP, SNP rs ID; Chr, chromosome; bp, physical position; refA, the effect allele; altA, the other allele; freq, frequency of the effect allele in the original data; b, se and p, effect size, standard error and P-value from the original (before COJO) GWAS; bJ, bJ_se and pJ, effect size, standard error and p-value from a joint analysis of all the selected SNPs; bC, bC_se, and bC_pval, effect size, standard error and P-value in the GWAS results conditioned on BMI (obtained with mtCOJO); Gene, closest gene to QTL; Gene location, gene location relative to QTL.

Title: Supplementary Data 4. FUMA gene-based analysis results

Description: Gene-based analysis results. Analysis were conducted with MAGMA v1.06, using default parameters (SNP-wide mean model) and with the 1000 Genome Phase3 selected as reference panel Columns are: GENE, gene Ensembl ID; CHR, chromosome; START/STOP, annotation boundaries of the gene on that chromosome; NSNPs, number of SNPs annotated to the gene that were found in the data and were not excluded based on internal SNP QC; NPARAM, number of relevant parameters used in SNP-wise (mean) model; N, sample size; ZSTAT, the Z-value for the gene, based on its (permutation) *P*-value (this is what is used as the measure of gene association in the gene-level analyses); P, the gene *P*-value, using asymptotic sampling distribution (if available); SYMBOL, Gene name.

Title: Supplementary Data 5. Results from FUMA gene set analysis

Description: Significant gene sets (after multiple testing correction) in the competitive gene-set analysis, containing output for the individual genes in the significant gene sets. Analysis were conducted with MAGMA v1.06. Columns are: GENE, gene Ensembl ID; CHR, chromosome; START/STOP, the annotation boundaries of the gene on that chromosome; NSNPS, the number of SNPs annotated to that gene that were found in the data and were not excluded based on internal SNP QC; NPARAM, the number of relevant parameters used in the model; N, the sample size used when analysing that gene; ZSTAT, the Z-value for the gene, based on its (permutation) *P*-value; P, the gene *P*-value, using asymptotic sampling distribution. Before each set, two general parameters are defined, namely ALPHA and NUMBER_OF_TESTS, denoting the nominal significance threshold and number of tests used to determine significance for the results shown. Additionally, each set is labelled with three tags: VARIABLE, name of the gene set; NGENES, number of genes in the data that are in the set; and P-VALUE, competitive gene-set *P*-value.

Title: Supplementary Data 6. FUMA results for annotation of genes to tissue enriched expression

Description: Tissue expression analysis. MAGMA gene-property test was performed for average gene-expression per category (e.g. tissue type or developmental stage) conditioning on average expression across all categories (one-side). Analysis were conducted with MAGMA v1.06. Columns are: VARIABLE, name of the gene set, gene covariate or interaction; TYPE, the type of variable, either SET or COVAR for normal gene sets and gene covariates provided in the input files; NGENES, the number of genes in the data that are in the set (for gene sets and set by covariate interactions), that are in the interaction set (for set by set interactions), or for which non-missing values were available (for gene covariates); BETA, the regression coefficient of the variable; BETA_STD, semi-standardized regression coefficient, corresponding to the predicted change in Z-value given a change of one standard deviation in the predictor gene set / gene covariate (ie. BETA divided by the variable's standard deviation); SE, standard error of the regression coefficient; P, *P*-value for the parameter / variable; FULL_NAME: the full variable name; only included if the variable names exceed the maximum length for the VARIABLE column. General parameters and settings for the analysis are included at the top. These are: TOTAL_GENES, the total number of genes included in the analysis; TEST_DIRECTION, the testing direction used for the different types of parameters; CONDITIONED_INTERNAL/RESIDUALIZED/HIDDEN/VARIABLES: the internal covariates and external variables which the analysis was conditioned on.

Title: Supplementary Data 7. SNP-based heritability analysis using annotations for cell types

Description: Heritability partitioned by cell type, using LD score regression (Finucane et al., 2015). Columns are: Name, cell type; Coefficient and Coefficient_std_error, regression coefficient standard

error of corresponding to the cell type specific annotation; Coefficient_P_value, *P*-value from a one-sided test that the coefficient is greater than zero.

Title: Supplementary Data 8. SNP-based heritability analysis using annotations for functional categories.

Description: Proportion of SNP-heritability and enrichment for 53 functional categories, estimated with LD score regrssion (Finucane et al., 2015). Associations highlighted in orange pass the significance threshold accounting for multiple testing (P < 9.4 x 10-4). Columns are: Category, functional annotation; Prop._SNPs, proportion of SNPs in the category; Prop._h2 and Prop._h2_std_error, proportion of heritability and respective standard error; Enrichment, enrichment of the category, defined asthe proportion of SNP heritability in the category divided by the proportion of SNPs in that category; Enrichment_std_error and Enrichment_p, standard error and *P*-value of the category enrichment.

Title: Supplementary Data 9. SMR significant associations in different tissues

Description: Genes associated with 25OHD through summary-data-based Mendelian randomization (SMR)(Zhu et al., 2016). Columns are: tissue, eQTL study/tissue where eQTL was identified; probeID, gene probe ID; Chr, probe chromosome; Probe_bp, probe physical position; Gene, gene name; topSNP, top associated cis-eQTL of the probe in the eQTL study; A1, effect (coded) allele; b_SMR, estimated effect size of gene expression on trait from the SMR method (bxy); se_SMR, standard error of bxy; p_SMR, *P*-value from the SMR test; p_HEIDI, P-value of the HEIDI test; b_GWAS, effect size of the top cis-eQTL in the GWAS; se_GWAS, standard error of the top cis-eQTL in th GWAS; p_GWAS, *P*-value of the top cis-eQTL in the GWAS; b_eQTL, effect size of the top cis-eQTL in the eQTL study; se_QTL, standard error of b_eQTL; p_eQTL, *P*-value of the top cis-eQTL in the eQTL study; N_probes, number of probes tested in the tissue; bonf_P, Bonferroni-corrected threshold for significance for that tissue.

Title: Supplementary Data 10. Genetic correlation between 25OHD and other traits

Description: Results are presented for 25OHD with no BMI correction and for two levels of BMI correction (BMIcov, with BMI as covariate; BMIcond, conditioned on BMI with mtCOJO). Genetic correlation (rg) was calculated between 25OHD and 764 traits (746 unique traits from LD Hub, and an additional 17 with GWAS summary statistics that are more recent than those included in LD Hub). Studies that were repeated in LDHub and had different estimates of rg were not included. There are more than 764 rows because, for some traits, LD Hub had GWAS results available from different studies (e.g. birth weight). Note that some traits have a significant LDSC intercept. These are traits measured in the UK Biobank, where sample overlap proportion is 1, hence intercept as expected from theory is the phenotypic correlation.

Columns are: PMID, PubMed ID of trait with which rg was calculated (summary statistics generated within the program of complex trait genomics (PCTG) are labelled "PCTG in-house analyses of UKB data using BOLT-LMM"); LDHub, whether GWAS summary statistics for trait2 were from LD hub; h2, observed scale h2 for trait2; rg, se, and p, genetic correlation and respective standard error and *P*-value; gcov_int and gcov_int_se, LDSC regression intercept and respective standard error.

Title: Supplementary Data 11. Bi-directional GSMR associations between vitamin D and other traits

Description: Mendelian randomisation analysis estimates from GWAS results from 25OHD and selected phenotypes, obtained with generalized summary mendelian randomization (GSMR)(Zhu et al., 2018).No results were available when the number of SNP instruments was lower than 10. Columns are: Exposure/Outcome, exposure and outcome traits tested; Heidi filtering, whether HEIDI-outlier filtering was applied; bxy, GSMR estimated effect size of the exposure on the outcome; se, standard error of bxy; p, *P*-value of bxy; nsnp, number of genetic instruments used in the GSMR analysis.

Title: Supplementary Data 12. 2SMR results for causal associations identified with GSMR

Description: To validate some of the causal associations identified with generalized summary mendelian randomization (GSMR), we ran 2-sample Mendelian randomization analyses (2SMR)(Hemani et al. 2018). For comparability, we used the same instruments that were used in the GSMR analyses. Note, however, that in some cases the number of instruments is lower in 2SMR because palindromic variants with intermediate allele frequencies are excluded with this method.

Title: Supplementary Data 13. 25 independent vQTLs

Description: Results are presented for the 25 most significant vQTLs identified as LD-independent through clumping (LD $r^2 < 0.01$).Highlighted in orange are vQTLs with a GWS ($P < 5x10^{-8}$) interaction with season. Association results (effect size, standard error and *P*-value) from the vQTL GWAS, QTL GWAS (full GWAS and season-stratified), and GxE with season are presented. Columns are: type, type of QTL; SNP, SNP rs ID; CHR, chromosome; BP, physical position; A1 and A2, effect allele and other allele, respectively; freq, frequency of the effect allele; NMISS, sample size in vQTL GWAS; BETA, SE, Z and P, effect size, standard error, z-score and *P*-value; Gene, closest gene to vQTL; Gene location, gene location relative to vQTL; PheWAS, traits with a GWS ($P < 5 \times 10^{-8}$) association reported for the vQTL SNP (information obtained from the Gene ATLAS online database (Canela-Xandri et al. 2018)).

Title: Supplementary Data 14. Association results from the GxE analysis with season

Description: Results are presented for variants with MAF > 0.05 and a GWS ($P > 5 \times 10^{-8}$) interaction with season. Highlighted in orange are the top independent (LD $r^2 < 0.01$) GxE associations. Association results (Z-score, effect size, standard error and *P*-value) from the GxE analysis with season, vQLT GWAS and season-stratified QTL GWAS (winter and summer) are presented. Columns are: SNP, SNP rs ID; CHR, Chromosome; BP, base pair; GxE with season Z and P, Z score and asymptotic *P*-value test for interaction term with season; indep_vQTL, whether the SNP was a lead SNP in the clumped vQTL results; vQTL GWAS, Winter GWAS and Summer GWAS BETA, SE, and P, effect size, standard error and *P*-values from are from the vQTL GWAS, and those with suffixes _winter and _summer are results from the season-stratified GWA analyses.
