# Supplementary material for: Genome-wide association study identifies 143 loci associated with 25 hydroxyvitamin D concentration
Source: Nat Commun. 2020 Apr 2;11:1647. doi: 10.1038/s41467-020-15421-7 (PMC7118120; doi:10.1038/s41467-020-15421-7)
Supplement: Supplementary file 17 — Reporting Summary [file 41467_2020_15421_MOESM17_ESM.pdf]

nature research

Corresponding author(s): John McGrath

Last updated by author(s): Mar 1, 2020

Reporting Summary

Nature Research wishes to improve the reproducibility of the work that we publish. This form provides structure for consistency and transparency in reporting. For further information on Nature Research policies, see Authors & Referees and the Editorial Policy Checklist .

Statistics

For all statistical analyses, confirm that the following items are present in the figure legend, table legend, main text, or Methods section.

n/a	Confirmed	
☐	☒	The exact sample size (*n*) for each experimental group/condition, given as a discrete number and unit of measurement
☐	☒	A statement on whether measurements were taken from distinct samples or whether the same sample was measured repeatedly
☐	☒	The statistical test(s) used AND whether they are one- or two-sided<br>*Only common tests should be described solely by name; describe more complex techniques in the Methods section.*
☐	☒	A description of all covariates tested
☐	☒	A description of any assumptions or corrections, such as tests of normality and adjustment for multiple comparisons
☐	☒	A full description of the statistical parameters including central tendency (e.g. means) or other basic estimates (e.g. regression coefficient) AND variation (e.g. standard deviation) or associated estimates of uncertainty (e.g. confidence intervals)
☐	☒	For null hypothesis testing, the test statistic (e.g. *F*, *t*, *r*) with confidence intervals, effect sizes, degrees of freedom and *P* value noted<br>*Give P values as exact values whenever suitable.*
☐	☒	For Bayesian analysis, information on the choice of priors and Markov chain Monte Carlo settings
☒	☐	For hierarchical and complex designs, identification of the appropriate level for tests and full reporting of outcomes
☐	☒	Estimates of effect sizes (e.g. Cohen's *d*, Pearson's *r*), indicating how they were calculated

Our web collection on statistics for biologists contains articles on many of the points above.

Software and code

Policy information about availability of computer code

Data collection	UK Biobank (UKB) data were obtained through direct application to the UK Biobank (full details are available in https://www.ukbiobank.ac.uk/). GWAS summary statistics used for the meta-analysis and mendelian randomization were publicly available (see “Data” section below) or PCTG in-house analyses of UKB data using fastGWA . All analyses and data security procedures were conducted under human research ethics approval from the University of Queensland. Functional annotations (including annotation elements constructed using ENCODE and Roadmap Epigenomics Consortium data) to partition SNP-based heritability with LDSC were downloaded from https://data.broadinstitute.org/alkesgroup/LDSCORE/.
Data analysis	Analyses were conducted with the following software: R version 3.5.0 , PLINK1.9, PLINK2, impG version 1.0.1, several tools implemented in GCTA 1.92.3beta3 (fastGWA, COJO, GSMR, mtCOJO, GREML-LDMS), OSCA version 0.45, GCTB (SBayesS and SBayesR), 2SMR version 0.4.26, LDSC, FUMA online platform.

For manuscripts utilizing custom algorithms or software that are central to the research but not yet described in published literature, software must be made available to editors/reviewers. We strongly encourage code deposition in a community repository (e.g. GitHub). See the Nature Research guidelines for submitting code & software for further information.

Data

Policy information about availability of data

All manuscripts must include a data availability statement. This statement should provide the following information, where applicable:

- Accession codes, unique identifiers, or web links for publicly available datasets
- A list of figures that have associated raw data
- A description of any restrictions on data availability

Full UKB data is available by direct application to the UK Biobank.The SUNLIGHT data were downloaded from https://drive.google.com/drive/folders/0BzYDtCo_doHJRFRKR0ltZHZWZjQ. Functional annotations to partition SNP-based heritability with LDSC were downloaded from https://data.broadinstitute.org/alkesgroup/LDSCORE/. eQTL data were downloaded from http://www.eqtlgen.org/cis-eqtls.html and https://cnsgenomics.com/software/smr/#DataResource. GWAS summary statistics used for bi-directional GSMR were downloaded from https://walters.psycm.cf.ac.uk (schizophrenia), https://

cnsgenomics.com/content/data (type II diabetes), https://ctg.cncr.nl/software/summary_statistics (Alzheimer's disease; fluid intelligence; ADHD), https://www.thessgac.org/data (educational attainment), https://www.med.unc.edu/pgc/download-results/ (bipolar disorder; autism spectrum disorder), http://plaza.umin.ac.jp/~yokada/datasource/software.htm (rheumatoid arthritis), ftp://ftp.ebi.ac.uk/pub/databases/gwas/summary_statistics/vanderHarstP_29212778_GCST005194 (coronary artery disease), https://www.ibdgenetics.org/downloads.html (inflammatory bowel disease). All other data are contained in the article and its supplementary information or are available on request

Field-specific reporting

Please select the one below that is the best fit for your research. If you are not sure, read the appropriate sections before making your selection.

- [x] Life sciences
- [ ] Behavioural & social sciences
- [ ] Ecological, evolutionary & environmental sciences

For a reference copy of the document with all sections, see nature.com/documents/nr-reporting-summary-flat.pdf

Life sciences study design

All studies must disclose on these points even when the disclosure is negative.

Sample size

We restricted our analysis to the 417,580 individuals in the UK Biobank with (1) European ancestry, (2) vitamin D 25OHD levels measurements, and (3) genotype data available. We did not perform sample-size calculations, but our sample had 99% power to detect loci with small effects (explaining 0.015% of the variance), at the genome-wide significance threshold of 5x10-8.

Data exclusions

We excluded individuals without genotype data or 25OHD measurements as these were required to conduct the genome-wide association (GWA) analyses. We also restricted our analyses to individuals of European ancestry as GWA analyses should be conducted within same ancestry groups (given the different linkage disequilibrium structures underlying different populations). We opted to analyse Europeans as this was the group with larger sample size in the UKB. These exclusion criteria were established before the study.

Replication

We examined the prediction of the polygenic risk score in two independent samples: (1) the QIMR Brisbane-based twin and family sample, and (2) an independent UKB sample of the same size as the QIMR sample. Polygenic score predictor using SNP effect weights estimated in the UKB explained up to 10.5% and 5.7% of variance (after accounting for covariates) in independent samples QIMR and UKB.

Randomization

not applicable

Blinding

not applicable

Reporting for specific materials, systems and methods

We require information from authors about some types of materials, experimental systems and methods used in many studies. Here, indicate whether each material, system or method listed is relevant to your study. If you are not sure if a list item applies to your research, read the appropriate section before selecting a response.

Materials & experimental systems

n/a	Involved in the study	
x		Antibodies
x		Eukaryotic cell lines
x		Palaeontology
x		Animals and other organisms
	x	Human research participants
x		Clinical data

Methods

n/a	Involved in the study	
x		ChIP-seq
x		Flow cytometry
x		MRI-based neuroimaging

Human research participants

Policy information about studies involving human research participants

Population characteristics	The UK Biobank is a well described sample. Participants were recruited between 2006 and 2010. Informed consent was obtained by UK Biobank for all participants, and the study was approved by the North West Multicentre Research Ethics Service Commitee.<br><br>The QIMR Brisbane-based twin and family samples (N = 1,632 unrelated) were collected between May 1992 and January 2014, mostly from South-East Queensland (latitude 27° S). Mean age (SD): 33.2 (15.5); gender: 850 female, 787 Male. All subjected were of European ancestry. Genotypes were obtained on Illumina Omni chips.
Recruitment	Details about the recruitment for the UK Biobank are well described on their website https://www.ukbiobank.ac.uk/<br><br>The primary subjects in the QIMR Brisbane-based twin and family sample were adolescent twins born between 1977 and 2003 (n = 3208) recruited for the Brisbane Longitudinal Twin Study on melanoma risk factors (Wright and Martin 2004, Zhu et al. 2007). Data collected from the twin pairs at approximately age 12, their siblings (within 5 years of the twins' age) and parents when possible. Samples were collected between May 1992 and January 2014, mostly from South-East Queensland (latitude 27° S). At this latitude, there is sufficient UVR to allow for vitamin D synthesis throughout the year
Ethics oversight	The UK Biobank study was approved by the North West Multicentre Research Ethics Service.<br><br>All procedures in the QIMR Brisbane-based twin and family sample collection were approved by the Human Research Ethics Committee of the QIMR Berghofer Medical Research Institute and were in compliance with the 1964 Helsinki declaration and its later amendments or comparable ethical standards. Legal guardians (as subjects were under the age of 18) gave written, informed consent prior to inclusion and testing for all participants.

Note that full information on the approval of the study protocol must also be provided in the manuscript.
